# The efficacy and safety of transcutaneous electrical nerve stimulation for labor analgesia in the first stage of labor: a qualitative and quantitative analysis

**DOI:** 10.3389/fmed.2026.1730360

**Published:** 2026-01-27

**Authors:** Zhuo-Ya Hu, Juan Tang, Xing-Xian Li, Dong-Ni Yuan, Zi-Yi Chen, Yang-Qian Lyu, Wen-Bin Ma, Lei Lan

**Affiliations:** 1 Acupuncture and Tuina College, Chengdu University of Traditional Chinese Medicine, Chengdu, China; 2 School of Health and Rehabilitation, Chengdu University of Traditional Chinese Medicine, Chengdu, China

**Keywords:** childbirth, labor analgesia, meta-analysis, systematic review, TENS, the first stage of labor, transcutaneous electrical nerve stimulation

## Abstract

**Aim:**

Labor pain represents a significant challenge for parturients during childbirth. Transcutaneous electrical nerve stimulation (TENS) is an effective analgesic modality. However, its efficacy and safety for intrapartum analgesia remain unclear. To address this knowledge gap, we performed a systematic review and meta-analysis of randomized controlled trials (RCTs) to comprehensively assess the analgesic effectiveness and safety profile of TENS during the first stage of labor.

**Methods:**

We searched databases from inception to October 17, 2024 and updated them to July 22, 2025. Paired researchers independently extracted data and assessed the risk of bias. All meta-analyses were performed via random effects models, and the GRADE approach was employed to evaluate the certainty of evidence.

**Results:**

We included 51 randomized controlled trials (10,038 participants, all females). Low evidence showed that compared with the blank control, parturients using TENS may experience more pain relief (WMD –1.98 cm, 95%CI -2.6 to −1.35 cm, the modelled RD 52, 95% CI 37 to 62%), parturients using TENS may shorten the duration of the first stage of labor (WMD –46.78 min,95% CI –61.32 to −32.25 min); compared with the epidural analgesia groups, parturients using TENS may shorten the duration of the first stage of labor (WMD-62.22 min, 95%CI -92.51 to −31.94 min).

**Conclusion:**

Compared with the blank control, TENS may reduce pain intensity and shorten the duration of the first stage of labor in parturients, with little to no difference in adverse events. When compared to epidural analgesia, TENS may shorten the duration of the first stage of labor, with very small differences observed in analgesic efficacy or adverse effects.

**Systematic review registration:**

https://www.crd.york.ac.uk/PROSPERO/view/CRD420251066439, identifier PROSPERO (CRD420251066439).

## 1 Introduction

Labor pain, which is primarily induced by uterine contractions and cervical dilation, represents one of the most intense pain experiences in a woman’s lifetime (1). Characterized by progressive intensification, it initially manifests as dull lower abdominal or lumbosacral discomfort, evolving into rhythmic and excruciating sharp pain as labor advances. The intensity of labor pain and the duration of labor are two critical clinical indicators during maternal delivery. Timely and effective analgesia is crucial for ensuring maternal safety, facilitating spontaneous vaginal delivery, and optimizing neonatal outcomes (2).

The following provides a description of Transcutaneous Electrical Nerve Stimulation (TENS): Its physiological basis encompasses (1) the Gate Control Theory, where stimulation of Aβ fibers inhibits nociceptive signal transmission (via Aδ and C fibers); (2) the release of endogenous opioids, elevating cerebrospinal fluid levels of *β*-endorphin; and (3) central nervous system inhibition via activation of descending pain inhibitory pathways. Its indications include conditions such as labor pain, low back pain, and neuropathic pain. Contraindications involve application over implanted electronic devices, the abdomen during pregnancy, the carotid sinus region, malignant tumor sites, and areas with bleeding tendencies. The most frequently reported side effects are skin irritation, manifested as erythema, pruritus, and discomfort beneath the electrodes. Key advantages are its non-invasive nature, safety, ease of administration, provision of immediate analgesia, and compatibility with other therapies. Its disadvantages include restricted efficacy against severe or deep-seated pain, significant inter-individual variability in therapeutic outcomes, and anatomical constraints on application. Epidemiologically, as a significant pain-relief modality, its utilization demonstrates an upward trend, with potential for reducing long-term healthcare expenditures. Compared to epidural analgesia, TENS is suitable for parturients who prioritize maintaining mobility, control, and a more natural birth experience. In contrast, epidural analgesia is indicated for those experiencing severe pain or those at high obstetric risk necessitating emergency cesarean section.

Currently, TENS is employed as a non-pharmacological approach for analgesia, with its non-invasive nature offering distinct clinical advantages (3). The review article by Lowe, titled “The Nature of Labor Pain” (4), systematically delineates the stages of labor, the characteristics and physiological mechanisms of pain, as well as analgesia strategies. However, the efficacy and safety of TENS for labor analgesia remain uncertain. The first stage of labor, the cervical dilation stage, extends from the onset of regular uterine contractions to complete cervical dilatation. Its analgesic significance lies in effectively blocking the pain of contractions, for which Transcutaneous Electrical Nerve Stimulation (TENS) can be employed. In contrast, the physiological focus of the second stage is fetal expulsion, where the pain transitions to somatic pain originating from the pelvic floor and perineum. Here, the analgesic goal shifts to balancing pain relief with the preservation of the parturient’s ability to push effectively, a balance often achieved using epidural analgesia. TENS is primarily indicated for the first stage and offers limited utility in the second stage, when investigating the analgesic efficacy of Transcutaneous Electrical Nerve Stimulation (TENS), the primary research focus is typically on the first stage of labor. This study aims to quantitatively evaluate the analgesic effects of TENS compared to blank control, and assess its benefits and limitations relative to epidural analgesia. The findings are expected to provide evidence-based guidance for clinical decision-making, particularly in resource-limited settings where the non-invasive characteristics of TENS may enhance its applicability.

## 2 Materials and methods

Our systematic review was conducted in accordance with the Preferred Reporting Items for Systematic Reviews and Meta-Analyses (PRISMA) guidelines (5) and was prospectively registered on PROSPERO (CRD420251066439).

### 2.1 Literature search

Researchers have developed database-specific search strategies, without language restrictions or publication status limitations, for Pubmed, EMBASE, Cochrane Central Register of Controlled Trials (CENTRAL), Web of Science, Chinese National Knowledge Infrastructure (CNKI), VIP Database for Chinese Technical Periodicals, and Wan Fang, which were searched from inception to October 17, 2024 and updated to July 22, 2025 (Supplementary Table 1). We also reviewed previous systematic reviews and included other studies that have met the criteria. We systematically examined the reference lists of prior systematic reviews (6–8) and cross-verified all potentially eligible randomized controlled trials (RCTs), implementing individual exclusion assessments.

### 2.2 Literature screen and data extraction

A pair of researchers, DNY and XXL, and ZYC and YQL, independently screened the titles and abstracts using a unified standard, and then the full texts that met the criteria were selected. Standardized pretest forms, with comprehensive instructions were used to ensure consistent application across all research sites. Any discrepancies between researchers were resolved through group discussion or, when necessary, with the assistance of an arbitrator (ZYH) to ensure consensus.

We included the following trials: (1) naturally delivered, full-term pregnancy (≥37 weeks), singleton mothers, aged more than 18 years old; (2) on the basis of usual care, TENS vs. blank treatment, or TENS vs. epidural stimulation; (3) pain intensity or duration reduction, during the first stage of labor; (4) Parallel-group randomized controlled trial.

A pair of reviewers: DNY and XXL, ZYC and YQL, independently abstracted data from each eligible trial. We have collected the relevant information of the article, including the author’s name and publication year, country of origin, sample size, characteristics of the parturient, intervention and outcome indicators, etc. We extracted the change scores from the baseline to reflect the internal changes in individuals.

### 2.3 Risk of bias assessment

Reviewers (ZYH, DNY and XXL) independently assessed the risk of bias, via a modified Cochrane Risk of Bias Tool 1.0 (9, 10), including random sequence generation, allocation concealment, blinding of study participants, operators, data collectors, evaluators and analysts, incomplete outcome data (≥20% missing data was considered high risk of bias), and other potential sources of bias. The answer options for each question are “definitely or possibly” (low bias risk) or “definitely or possibly Not” (high bias risk). Disagreements among researchers are resolved through discussion, those that cannot be resolved are determined by a third party.

### 2.4 Data analysis

We computed relative risk (RR) with 95% confidence intervals for dichotomous outcomes, while weighted mean differences (WMD) with 95% CIs were derived for continuous variables. We converted all continuous result data to a common scale in each domain (11): (1) pain intensity to the 10 cm visual analogue scale (VAS) for pain, the minimal clinically important difference (MID) was 1 cm (12, 13). (2) Duration of the first stage of labor. Modeled values were computed to facilitate comparative interpretation of the results.

All meta-analyses were performed via a Der Simonian-Laird random-effects model, with statistical processing executed in STATA 17 (Stata Corp, College Station, TX, USA).”

We examined publication bias through the visual assessment of funnel plot asymmetry when 10 or more studies were included in the analysis (14). All comparisons were two-tailed using a threshold of *p* ≤ 0.05.

### 2.5 Certainty of evidence

The certainty of evidence for each outcome was evaluated via the GRADE (Grading of Recommendations, Assessment, Development, and Evaluation) methodology (15, 16). While randomized controlled trials initially receive high certainty ratings, downgrading may occur across five domains: risk of bias, consistency, directness, precision, and publication bias, potentially yielding moderate, low, or very low certainty assessments. Treatment effects were judged to be imprecise under two distinct conditions: (1) for pain outcomes, when the 95% confidence interval encompassed half of the minimal clinically important difference (MID); (2) for adverse events, when the 95% CI included the null effect value (Table 1).

**Table 1:** Baseline characteristics of included studies.

Study ID	Intervention	Control	Funding	Country	Number of participants at baseline, n	Mean duration of gestational age (SD), weeks	Mean age (SD), years
Gao Y 2023(17)	TENS	Blank	NR	China	160	39.7 (0.95)	27.8 (2.22)
M. Movahedi 2022(64)	TENS	Blank	NR	Iran	100	38.9 (0.81)	29.9 (4.12)
Gao XX 2021(18)	TENS	Blank	NR	China	220	39.2 (1.42)	30.3 (4.08)
Yan J 2021(19)	TENS	Blank	NR	China	286	39.7 (0.84)	26.74 (2.79)
A. Njogu 2021(20)	TENS	Blank	governmental	China	413	39.1 (0.89)	29 (3.52)
Lei FY 2021(21)	TENS	Blank	NR	China	140	39.7 (1.65)	28.5 (4.69)
Peng LL 2021(22)	TENS	Blank	NR	China	145	39.1 (1.3)	27.8 (2.42)
Zhang XF 2020(23)	TENS	Blank	NR	China	200	NR	28.37 (4.16)
Zhang LQ 2020(24)	TENS	Blank	NR	China	86	NR	28.9 (3.44)
Li HY 2020(25)	TENS	Blank	governmental	China	100	38.7 (1.13)	29.6 (2.74)
Huang JZ 2020(26)	TENS	Blank	NR	China	160	39.4 (0.96)	28.7 (7.33)
Liu PP 2020(27)	TENS	Blank	NR	China	186	38.7 (1.18)	28 (1)
Jiang DM 2020(28)	TENS	EA	NR	China	50	39.5 (0.56)	29.6 (0.92)
Huang LY 2019(29)	TENS	EA	NR	China	480	NR	26.4 (2.25)
Zhao ZP 2018(30)	TENS	Blank	NR	China	92	38.2 (0.72)	28.3 (3.34)
A. Baez-Suarez 2018(62)	TENS	Blank	NR	Spain	63	39.5 (1.42)	28.1 (5.51)
Lu L 2018(31)	TENS+EA	EA	governmental	China	200	NR	28.2 (3.64)
Li L 2018(32)	TENS	Blank	governmental	China	80	38.5 (1.49)	29 (1.42)
A. Nyambura 2017 (33)	TENS	Blank	NR	China	326	39.1 (0.89)	29 (3.52)
Liu J 2016(34)	TENS	Blank	NR	China	100	39 (1.14)	27.4 (3.44)
	TENS	EA	NR	China	100	38.9 (1.1)	27.6 (3.61)
Xiao H 2015(35)	TENS+EA	EA	governmental	China	40	NR	NR
Li J 2015(36)	TENS	EA	government	China	80	NR	NR
	TENS	Blank	governmental	China	80	NR	NR
Cai XL 2015(37)	TENS	Blank	NR	China	172	NR	NR
	TENS	EA	NR	China	172	NR	NR
Xiao H 2015(38)	TENS + EA	EA	governmental	China	40	39.3 (1.55)	26.6 (2.27)
Li HY 2012(39)	TENS	Blank	NR	China	60	38.6 (NR)	28.4 (NR)
Xu MJ 2006 (39)	TENS	Blank	NR	China	60	39.3 (0.64)	29.1 (3.58)
	TENS	EA	NR	China	60	39.3 (0.7)	28.8 (3.6)
Su XJ 2001(41)	TENS	Blank	NR	China	40	39.3 (0.5)	25.8(0.96)
Yang X 2021(42)	TENS	Blank	NR	China	60	40 (0.47)	28(4.06)
An ZZ 2015(43)	TENS	Blank	NR	China	60	NR	NR
Cao JG 2025(44)	TENS	Blank	governmental	China	136	39.1 (1.12)	27.6 (5.84)
Wang L 2019(45)	TENS	Blank	NR	China	64	39.2 (0.7)	28.6 (4.3)
Xu JH 2022(46)	TENS	Blank	governmental	China	60	39.1 (1.12)	27.6 (5.84)
Song KK 2023(47)	TENS	Blank	governmental	China	90	38.7 (0.95)	30 (3.15)
He J 2020(48)	TENS	Blank	NR	China	200	39.1 (1.1)	28.5 (8)
Ma ZH 2018(49)	TENS	Blank	NR	China	60	37.5 (3.81)	27.5 (6.05)
Miao WJ 2020 (50)	TENS	EA	NR	China	151	39.6 (0.7)	27.5 (3.03)
Meng LK 2020(51)	TENS	Blank	NR	China	130	38.6 (1.26)	28.8 (4.27)
Han CP 2021(52)	TENS	EA	governmental	China	200	38.5 (1.15)	30.4 (3.3)
Zhao KL 2024(53)	TENS	Blank	governmental	China	130	39.6 (0.91)	27.1 (5.74)
Shi J 2002(54)	TENS	Blank	NR	China	60	NR	NR
Niu CY 2017(65)	TENS	Blank	NR	China	400	NR	22.3 (4.63)
Liu Ye 2015(55)	TENS	EA	NR	China	60	39.2 (0.76)	27.8 (3.93)
	TENS	Blank	NR	China	60	39 (0.75)	27.1 (3.9)
QianJ 2025(56)	TENS	Blank	governmental	China	60	40.2 (1.82)	31.5 (1.62)
Miao Y 2025(57)	TENS	EA	governmental	China	92	NR	NR
Xu J 2024(58)	TENS	Blank	NR	China	60	NR	30.3 (4.53)
Shi XL 2024(59)	TENS	Blank	governmental	China	80	39.6 (1.03)	26.4 (3.66)
	TENS	EA	governmental	China	80	39.6 (1.05)	26.4 (3.64)
Huang XZ 2019(66)	TENS	Blank	NR	China	94	39.3 (0.72)	28.8 (2.49)
R. Sulu 2022(61)	TENS	Blank	governmental	Turkey	42	38.27 (0.55)	22.02 (3.13)
Santana, L. S.2016(60)	TENS	Blank	NR	Brazil	46	NR	20 (4)
Zahra MEHRI 2022(67)	TENS	Blank	NR	Iran	130	39 (1.32)	24.5 (4.1)
V. Rashtchi 2022(68)	TENS	Blank	NR	Iran	80	NR	24(4.16)

NR, not reported; TENS, transcutaneous electrical nerve stimulation; EA, epidural analgesia.

## 3 Results

### 3.1 Literature screening

We screened 3,905 citations, and 51 RCTs (7,096 participants) were ultimately included. Workflow is shown in Supplementary Figure 1.

### 3.2 Characteristics of included studies

Forty-four studies were conducted in China (17–59), three in Iran (60), one in Spain (31), one in Turkey (61), and one in Brazil (60). All participants were adult females, with a full-term (≥37 weeks) singleton delivery. The median of the mean age of participants was 26.5 years (IQR 18 to 35 years). Pain intensity were evaluated in 36 trials (19, 21, 24–29, 31–33, 36–42, 62, 63). First-stage labor duration was assessed in 33 trials (17–31, 33, 34, 36, 37, 39, 40, 43–47, 54–57, 59, 64, 65). Forty-five trials compared TENS with a blank control (17–27, 30–34, 36–49, 51, 53–56, 62–65), and eleven trials compared TENS with epidural analgesia (28, 29, 34, 36, 37, 40, 50, 54, 55, 57, 59). The TENS parameters for each article were also recorded (Table 4).

### 3.3 Risk of bias

All 51 trials had at least one risk-of-bias domain: 33 trials (66%) adequately generated random sequences, 4 trials (8%) concealed allocation, 3 trials (6%) blinded participants, 1 trial (2%) blinded healthcare providers, 3 trials (6%) blinded each of the data collectors, outcome assessors, and data analysts, and 2 trials (4%) had ≥20% data missing in the trial report (Supplementary Table 2).

### 3.4 TENS vs. blank control

#### 3.4.1 Pain intensity in the first stage of labor

**Figure 1:**
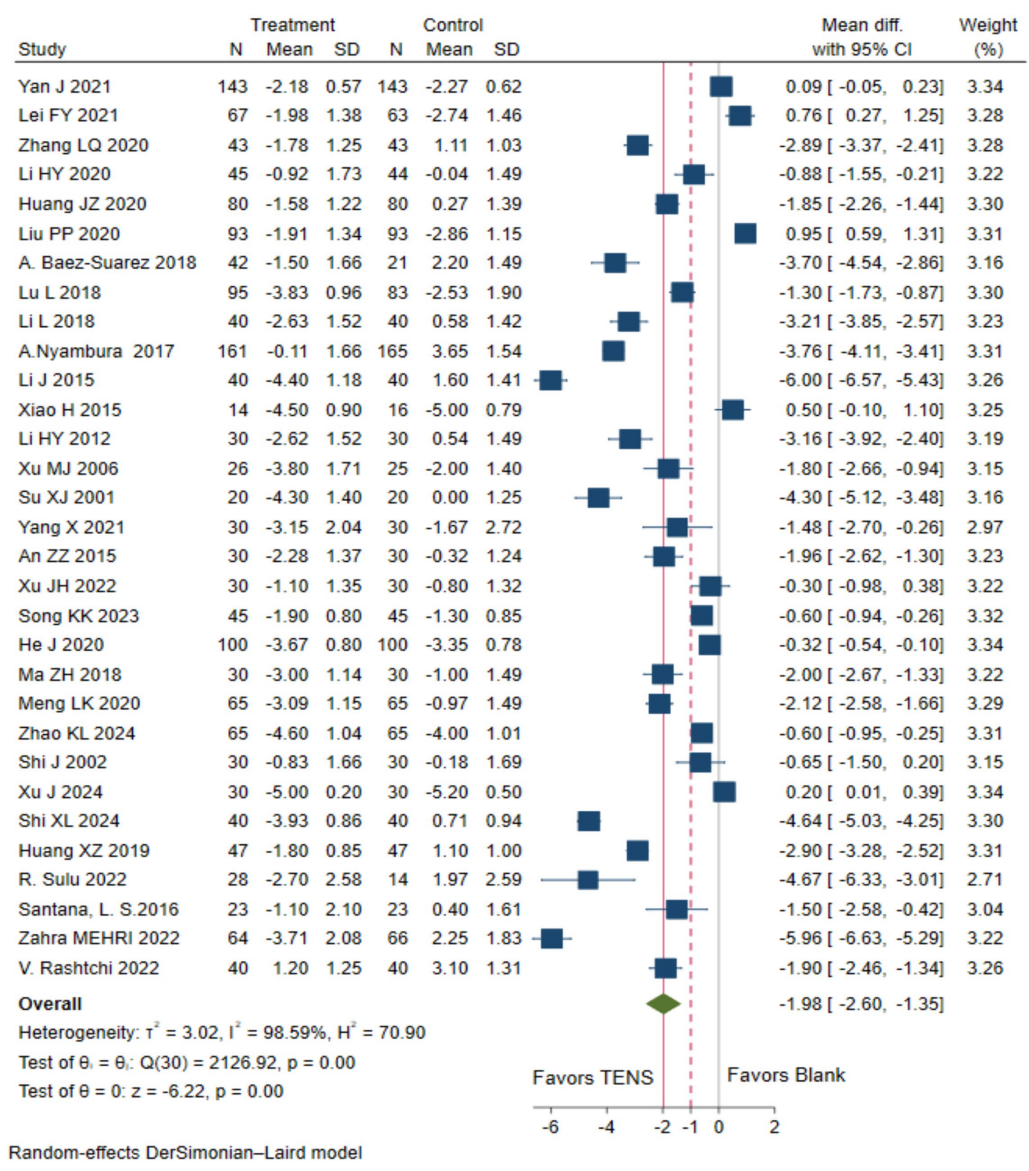
The pain intensity measured by the 10 cm VAS during the first stage of labor with TENS analgesia was compared to blank control.

Low evidence (31 RCTs, 3,227 patients) showed that compared with the blank control, parturients using TENS may experience more pain relief (WMD-1.98 cm, 95%CI -2.6 to −1.35 cm, modelled RD 52, 95%CI 37 to 62%; Table 2 and Figure 1) (19, 21, 24, 25, 27, 31–33, 36, 38, 41–43, 46–49, 51, 62, 63).

**Table 2:** Grade evidence profile of TENS versus the blank control for first-stage labor analgesia.

No. of trials (No. of patients)	Risk of bias	Inconsistency	Indirectness	Imprecision	Publication bias	Treatment association (95% CI)		Overall quality of evidence
Pain: 0–10 cm VAS for pain; lower is better; MID = 1 cm								
31 (3,227)	Serious ^a^	Not serious ^b^	Not serious	Not serious	Serious ^c^	Achieved at or above MID		Low
						TENS 79.6%	Control 27.9%	
						Modelled RD 0.52 (0.37,0.62)		
						WMD –1.98 cm (−2.6, −1.35 cm)		
Duration of the first labor stage(minutes): shorter is better								
30(4,092)	Serious ^a^	Not serious ^b^	Not serious	Not serious	Serious ^c^	WMD -46.78 min (−61.32, −32.25 min)		Low
Adverse effects								
11 (1,278)	Serious ^a^	Not serious, *I*^2^ = 27.5%	Not serious	Not serious	Serious ^c^	RR 0.51(0.38,0.69)		Low

NA, not available; MID, minimal clinical important difference. a, high risk of bias in blinding; b, The observed high variance originated primarily from substantial dispersion in reported treatment effects across studies, rather than data sparsity; c, high risk in publication bias.

#### 3.4.2 Duration of the first stage of labor

**Figure 2:**
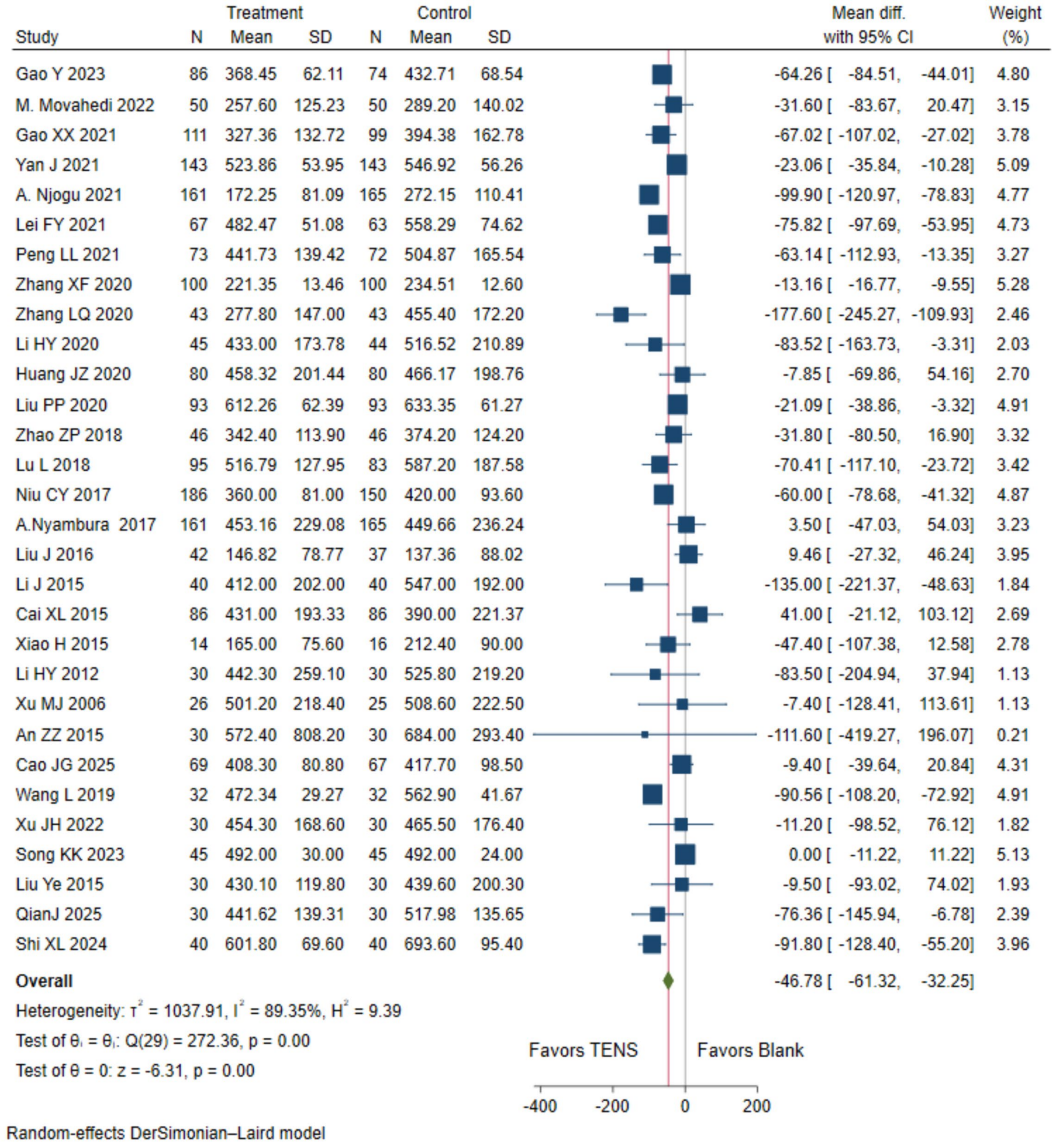
The duration of the first stage of labor with TENS analgesia was compared to blank control.

Low evidence (30 RCTs, 4,092 patients) reported that compared with blank control, parturients using TENS may shorten the duration of the first stage of labor (WMD-46.78 min,95%CI -61.32 to −32.25 min; Table 2 and Figure 2) (17–27, 30, 31, 33, 34, 36–40, 43–47, 55, 56, 58, 64, 65).

#### 3.4.3 Adverse effects

**Figure 3:**
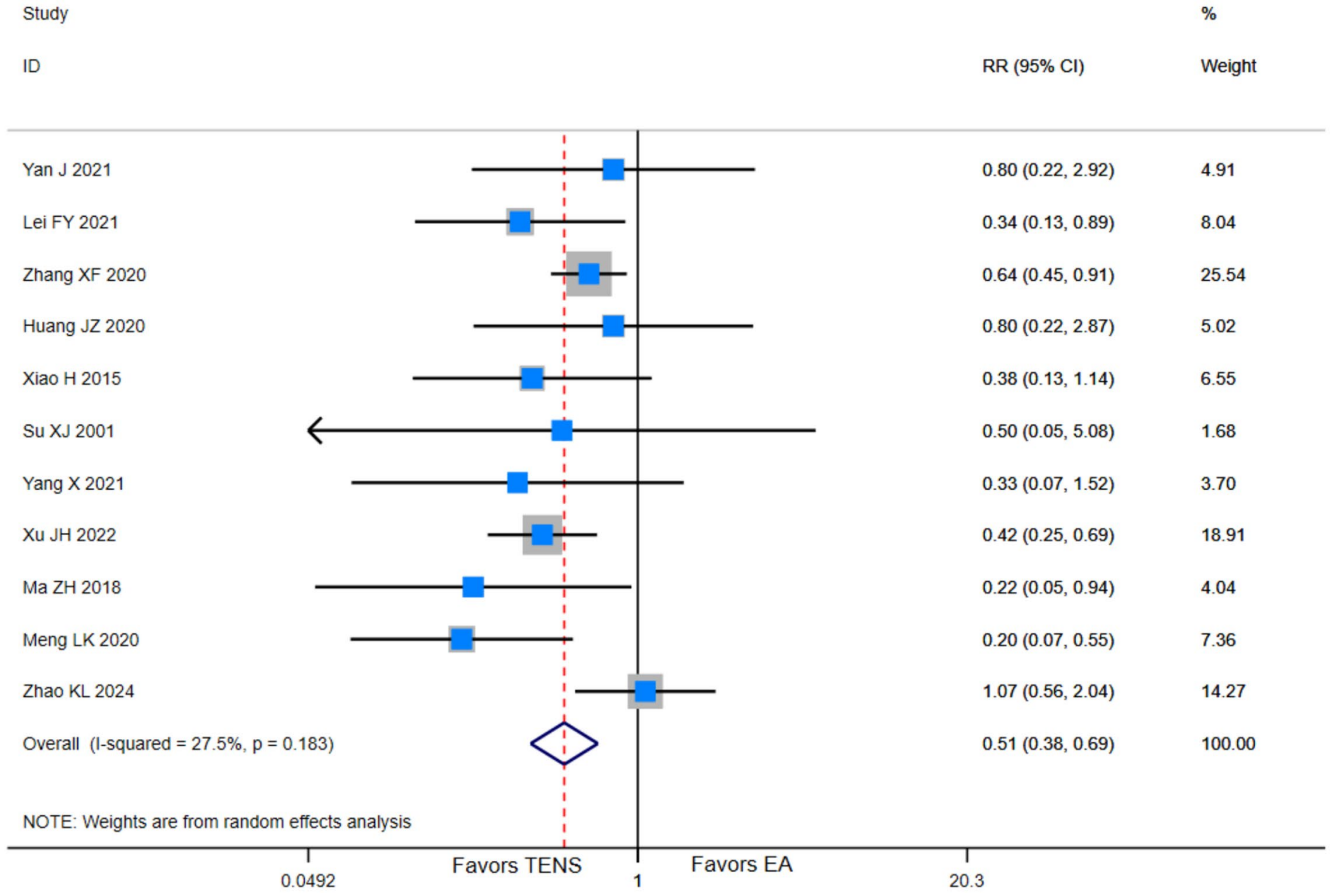
Adverse events among parturients in the first stage of labor who received TENS versus blank control.

Low evidence (11 RCTs,1,278 patients) suggests that compared with blank control, TENS may have little to no effect on reducing adverse effects in parturients during the first stage of labor (RR 0.51, 95% CI 0.38 to 0.69; Table 2 and Figure 3) (19, 21, 23, 26, 38, 41, 42, 46, 49, 51, 53).

### 3.5 TENS vs. epidural analgesia

#### 3.5.1 Pain intensity in the first stage of labor

**Figure 4:**
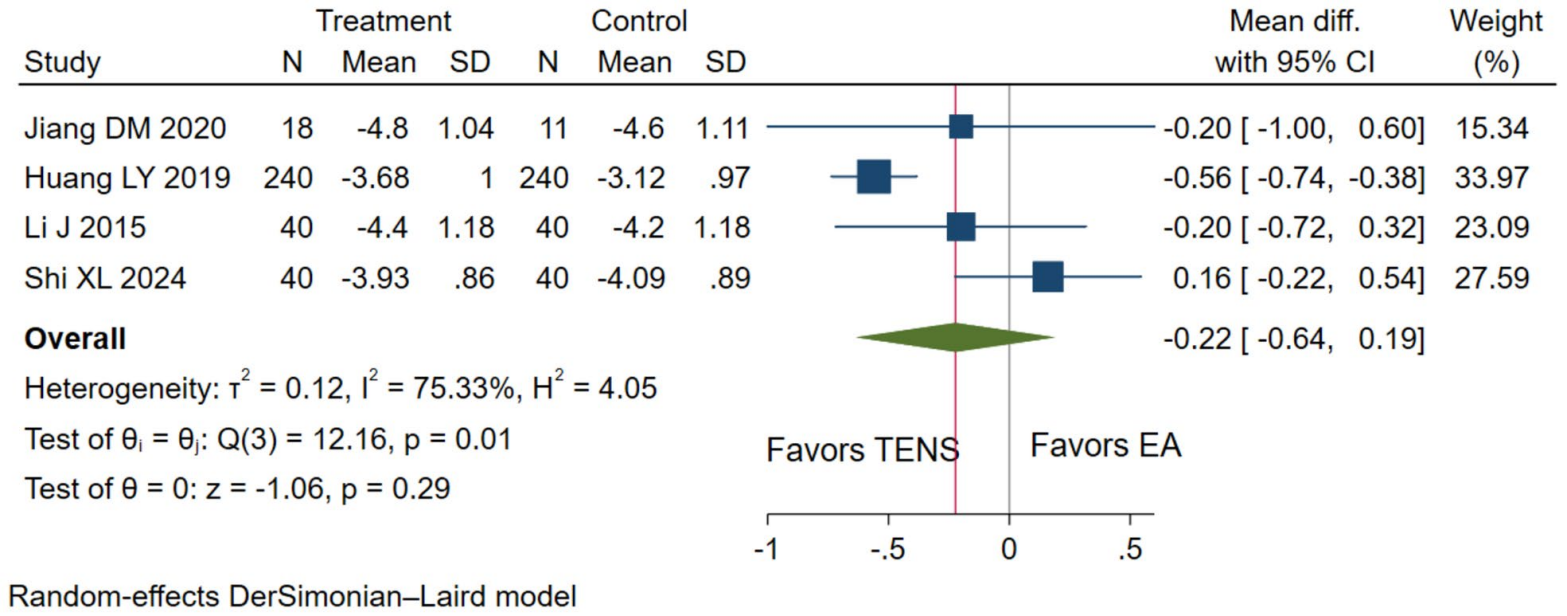
The pain intensity measured by the 10 cm VAS during the first stage of labor with TENS analgesia was compared to epidural analgesia.

Low evidence (4 RCTs, 669 patients) showed that compared with epidural analgesia groups, parturients using TENS may have little to no difference in pain relief (WMD-0.22 cm, 95%CI-0.64 to-0.19 cm, modelled RD 0, 95%CI 0 to 0%; Table 3 and Figure 4) (28, 29, 36).

**Table 3:** Grade evidence profile of TENS versus EA for first-stage labor analgesia.

No. of trials (No. of patients)	Risk of bias	Inconsistency	Indirectness	Imprecision	Publication bias	Treatment association (95% CI)		Overall quality of evidence
Pain: 0 to 10 cm VAS for pain; lower is better; MID = 1 cm								
4 (669)	Serious ^a^	Serious, *I*^2^ = 75.33%	Not serious	Not serious	NA	Achieved at or above MID		Low
						TENS 99.9%	Control 99.9%	
						Modelled RD 0.00 (0.00, 0.00)		
						WMD -0.22 cm (−0.64, 0.19 cm)		
Duration of the first labor stage(minutes): shorter is better								
8 (1,055)	Serious ^a^	Serious, *I*^2^ = 65.53%	Not serious	Not serious	Not serious	WMD -62.22 min (−92.51, −31.94 min)		Low
Adverse effects								
3 (271)	Serious ^a^	Not serious, *I*^2^ = 30.7%	Not serious	Serious ^b^	NA	RR 0.37(0.12,1.14)		Low

NA, not available. a, high risk of bias in blinding; b, small sample size.

**Table 4:** TENS Parameters.

Study ID	Frequency	Stimulation site	Device type	Intensity	Duration
Gao Y 2023(17)	2 Hz/100 Hz	Bilateral Zusanli (ST36), Sanyinjiao (SP6), Hegu (LI4)	Huatuo SDZ-II	15 mA	Until the end of the second stage of labor
M. Movahedi 2022(64)	100 Hz	Spinal nerve roots T10-L1 and S2-S4	Not specified	10–18 mA	30 min
Gao XX 2021(18)	Self-adjusted	Bilateral Hegu (LI4), median nerve 4 cm proximal to wrist crease; proximal to wrist crease; highest point of iliac crest to lumbar spinous process	China Doule Group GT500 Series Seventh Generation Multi-functional DAOLE Doule Instrument	Mild muscle tremor induced	Self-adjusted usage time
Yan J 2021(19)	Not specified	Bilateral Zusanli (ST36), Hegu (LI4), Sanyinjiao (SP6)	Not specified	Gradually increased from 15 mA to obvious tremor	20 min/session, twice, 2-h interval, Stop when the cervix is fully dilated. M
A. Njogu 2021(20)	Adjusted per tolerance	Hegu (LI4), Neiguan (PC6), paravertebral regions T10-L1 and S2-S4	SRL998A Bio-feed TENS System	Peak current: 15 mA	From the onset of the active phase until the end of the second stage of labor
Lei FY 2021(21)	2 Hz/100 Hz	Bilateral Hegu (LI4), Sanyinjiao (SP6), Zusanli (ST36)	Huatuo brand SDZ-II electronic acupuncture stimulator	Start at 15 mA, increase to slight muscle tremor and the pregnant woman’s tolerance level	Until the end of the second stage of labor
Peng LL 2021(22)	2 Hz	Bilateral Hegu (LI4), Sanyinjiao (SP6), Zusanli (ST36)	Huatuo brand SDZ-II electronic acupuncture stimulator	Start at 15 mA, increase to painless	Every 2 h for 30 min, until active phase
Zhang XF 2020(23)	Not specified	T10-L1, S2-S4, Bilateral Hegu (LI4), Neiguan (PC6)	Lebeier BTX-9800D labor analgesia instrument	Start at 15 mA, increase to distinct tremor without pain	Until full cervical dilation
Zhang LQ 2020(24)	2 Hz/100 Hz	Bilateral Hegu (LI4), Sanyinjiao (SP6), Zusanli (ST36)	SRL998 Youbeibei A type labor monitoring analgesia instrument	Start at 15 mA, increase to tolerable distinct tremor	Until the end of labor
Li HY 2020(25)	Not specified	Bilateral Hegu (LI4), Neiguan (PC6); Qihaishu (BL24), Guanyuanshu (BL26)	LABOUR-RI-Z TENS device	Not specified	Until entering the second stage
Huang JZ 2020(26)	40–80 Hz	Bilateral Hegu (LI4), DaLing (PC8); Paravertebral region of lower back	Lebeier labor analgesia instrument	Frequency: 0–55 mA. Usually, during contractions it is 25–40 mA, and during the intervals between contractions it is 5–10 mA.	Until full cervical dilation
Liu PP 2020(27)	Not specified	T10-L1, S1-S4	Fanlesheng non-invasive labor analgesia instrument	15–50 mA, to slight muscle tremor and the pregnant woman’s tolerance level	Until the end of the second stage
Jiang DM 2020(28)	Not specified	Bilateral Hegu (LI4), Neiguan (PC6), T10–L1, L5–S4	Not specified	Gradually increase to tolerance, stop at slight tremor	Not specified
Huang LY 2019(29)	2 Hz	Bilateral Hegu (LI4), Sanyinjiao (SP6), Zusanli (ST36)	Not specified	Start at 15 mA, increase to distinct tremor without pain and the pregnant woman’s tolerance level	30 min/session, 1 h interval, until active phase
Zhao ZP 2018(30)	Not specified	Both shoulders and lower back	“Lucky Baby” low-frequency nerve and muscle stimulator	It is advisable to use a mild tremor of the muscles and one that the pregnant woman can tolerate.	Until the end of the second stage
A. Baez-Suarez 2018(62)	100 Hz (TENS1); 80–100 Hz (TENS2)	Paravertebral regions T10–L1 and S2–S4	Cefar Rehab 2pro® TENS device	Individually titrated	30 min/session, may be extended
Lu L 2018(31)	2 Hz	Bilateral Hegu (LI4), Sanyinjiao (SP6), Zusanli (ST36)	Huatuo brand SDZ-II electronic acupuncture stimulator	Start at 15 mA, increase to distinct tremor without pain	30 min/session, 1 h interval, until active phase
Li L 2018(32)	2 Hz/100 Hz	Bilateral Hegu (LI4), Sanyinjiao (SP6), Zusanli (ST36)	Huatuo brand SDZ-II electronic acupuncture stimulator	Start at 15 mA, increase to distinct tremor and one that the pregnant woman can tolerate.	Until the end of the first stage
A. Nyambura 2017 (33)	Not specified	Bilateral Hegu (LI4), Neiguan (PC6); T10–L1, S2–S4	SRL998A labor monitoring analgesia instrument	Initial <30 units, then adjusted to tolerance, throughout labor, intensity 30–60 units	Until the end of labor
Liu J 2016(34)	active phase: 100 Hz; incubation phase: 3–10–100 Hz	T10–L1, S1–S4	KD-2A labor analgesia instrument	Not specified	30 min/session, 2 h interval
	2 Hz	Bilateral Hegu (LI4), Sanyinjiao (SP6)	KD-2A transcutaneous nerve stimulator	Start at 15 mA, increase to distinct tremor without pain	20 min/session, 2 h interval, until full dilation
Xiao H 2015(35)	1–100 Hz adaptive	Bilateral Hegu (LI4), Neiguan (PC6); T10–L1, L5–S4	Sanrui SRL998A biofeedback labor analgesia doula instrument	Peak output ≤15 mA	Not specified
Li J 2015(36)	Not specified	Bilateral Hegu (LI4), Neiguan (PC6); T10–L1, L5–S4	Not specified	Increase to tolerance, slight tremor	Not specified
	2 Hz	Bilateral Hegu (LI4), Sanyinjiao (SP6)	KD-2A transcutaneous nerve stimulator	Start at 15 mA, increase to distinct tremor without pain	20 min/session, 2 h interval, until full dilation
Cai XL 2015(37)	Not specified	Bilateral Hegu (LI4), median nerve 4 cm proximal to wrist crease; proximal to wrist crease; T10 (3 cm away from the left and right sides of the spine),5 cm vertically downward from the front side.	Not specified	Adjust to slight muscle tremor and tolerance	Stop after the second stage of labor or before a cesarean section; stop after the second stage of labor and before switching to a cesarean section to terminate natural childbirth
	2 Hz/100 Hz	Jiaji points (T10–L3), Ciliao points (S2–S4)	Han’s Acupoint Nerve Stimulator	15 ~ 25 mA	Once per hour for 30 min
Xiao H 2015(38)	2 Hz/100 Hz	Bilateral Hegu (LI4), Zhiyang (GV9, between T7-T8), Jizhong (GV6, between T11-T12)	Han’s Acupoint Nerve Stimulator	Hegu 8–12 mA, Back points 15–25 mA	Once per hour for 30 min
Li HY 2012(39)	Not specified	Bilateral Hegu (LI4), Neiguan (PC6), Shangliao (BL31), Ciliao (BL32), Zhongliao (BL33), Xialiao (BL34)	Not specified	Adjust to tolerance every 10 min	Until the end of delivery
Xu MJ 2006 (40)	2–100 Hz dense-dispersed wave	Jiaji points (T10–L1, L2 3 centimeters lateral to both sides of the spine), Ciliao points (S2–S4 offset 3 cm to the side)	Electronic acupuncture stimulator (Suzhou Medical Supplies Co., Ltd.)	15–30 mA, up to maximum tolerance	Once per hour for 30 min, until the end of the second stage
	2/100 Hz	Bilateral Hegu (LI4), Zusanli (ST36), Sanyinjiao (SP6)	Han’s Acupoint Nerve Stimulator	10–20 mA, to tolerance limit	Once per hour for 30 min, until delivery ends
Su XJ 2001(41)	Not specified	Bilateral Tianzong (SI11), Shenshu (BL23), Sanjiaoshu (BL22), Jianjing (GB21)	Fanke P0-9632 multifunctional electrotherapy apparatus	Adjust clockwise to tolerance	Until the end of delivery
Yang X 2021(42)	2 Hz	Bilateral Zusanli (ST36), Hegu (LI4), Sanyinjiao (SP6)	Huatuo brand electronic acupuncture stimulator	Start at 15 mA, increase to distinct tremor without pain	20 min/session, 2 h interval, until placental delivery
An ZZ 2015(43)	2 Hz/100 Hz	Bilateral Hegu (LI4), Sanyinjiao (SP6), Zusanli (ST36)	Not specified	Slowly increase to mild numbness/prickling	30 min/session, 2 h interval, until full dilation
Cao JG 2025(44)	2 Hz/100 Hz	Bilateral Hegu (LI4), Sanyinjiao (SP6)	Not specified	15–30 mA	Once per hour for 30 min, until 3 cm dilation
Wang L 2019(45)	2 Hz	Bilateral Zusanli (ST36), Hegu (LI4), Sanyinjiao (SP6)	Huatuo brand SDZ-II electronic acupuncture stimulator	Start at 15 mA, increase to distinct tremor without pain	20 min/session, 2 h interval, until the end of the second stage
Xu JH 2022(46)	2 Hz/100 Hz	Bilateral Hegu (LI4), Neiguan (PC6); Jiaji (T10–L1 3 centimeters lateral to both sides of the spine), Ciliao (S2–S4)	SRL998K fetal monitor/neuromuscular stimulator	15–50 mA, manual or auto adjustment	Not specified
Song KK 2023(47)	2 Hz	Bilateral Hegu (LI4), Zusanli (ST36), Sanyinjiao (SP6)	Huatuo brand SDZ-II electronic acupuncture stimulator	15–25 mA, increase to distinct tremor	20 min/session, 2 h interval, until the end of the second stage
He J 2020(48)	Hand: 10–30 Hz; Back: 20–40 Hz	Bilateral Hegu (LI4), Neiguan (PC6) (hands); Bilateral Shenshu (BL23), Sanjiaoshu (BL22), Eight Liao points (back)	SRL998A nerve and muscle stimulator	Hand: 6–20 mA; Back: 7–26 mA	Until the end of delivery
Ma ZH 2018(49)	2 Hz/100 Hz	Bilateral Hegu (LI4), Sanyinjiao (SP6), Zusanli (ST36)	Not specified	Start at 15 mA, increase to tolerable distinct tremor	Until fetal delivery
Miao WJ 2020 (50)	Not specified	Jiaji points (T10–L3 3 cm lateral to the spine (bilaterally).), Ciliao points (S2–S4 3 cm lateral to the spine (bilaterally).)	Korean-style nerve electrical stimulation analgesic device (HA NS)	Gradually increase to maximum tolerance	Not specified
Meng LK 2020(51)	2 Hz/100 Hz	Jiaji points (T10–L3), Ciliao (BL32)	Korean-style nerve electrical stimulation analgesic device (HA NS)	15–30 mA	30 min/session
Han CP 2021(52)	Not specified	Back: T10–L1, L5–S4; Hands: Bilateral Hegu (LI4), Neiguan (PC6)	SRL998A neuromuscular stimulator	Back: 30–70, Hand: 30–50 (units not specified)	From 3 cm to 10 cm cervical dilation
Zhao KL 2024(53)	Hand: 6–20 mA; Back: 20–40 Hz	Bilateral Hegu (LI4), Neiguan (PC6), Quchi (LI11) (hands); The horizontal line at the waist is the apex of the gluteal cleft, and the vertical axis is the spine.	Not specified	Hand: 6–20 mA, Back: 20–40 Hz	Until the end of delivery
Shi J 2002(54)	Not specified	Bilateral Hegu (LI4), Neiguan (PC6) (hands); T10–L1 (spine)	Lebeier labor analgesia instrument	Start at 15 mA, increase to muscle tremor without pain	Until full cervical dilation
Niu CY 2017(65)	2 Hz/100 Hz	Bilateral Hegu (LI4), Zusanli (ST36)	Not specified	Start at 15 mA, increase to distinct tremor without pain	30 min/session, 1 h interval, until full dilation
Liu Ye 2015(55)	2 Hz/100 Hz	Zusanli (ST36), Hegu (LI4), Sanyinjiao (SP6)	Not specified	Start at 15 mA, increase to tolerable distinct tremor, ≤30 mA	Once per hour for 30 min, until fetal delivery
	2 Hz/100 Hz	Bilateral Hegu (LI4), Sanyinjiao (SP6), Zusanli (ST36)	Not specified	Start at 15 mA, increase to tolerable distinct tremor	Until the end of the first stage
QianJ 2025(56)	100 Hz (TENS1); 80–100 Hz (TENS2)	Paravertebral regions T10–L1 and S2–S4	Cefar Rehab 2pro® TENS device	Not specified	30 min/session
Miao Y 2025(57)	100 Hz	1 cm lateral to the spine (bilaterally). T10–L1 and S2–S4	Portable TENS unit	Individually titrated	30 min/session
Xu J 2024(58)	2–4 Hz (Phase 1:until 8 cm dilation.); 100 Hz (Phase 2 until delivery end)	Phase 1(the cervix has dilated to 4 centimeters.): Sanyinjiao (SP6) Neiguan (PC6) (right hands); Phase 2(8 cm dilation to delivery end)Bilateral Hegu (LI4), Shenmen(HT7)	Portable TENS device	Gradually increase to tolerance	Until the end of delivery
Shi XL 2024(59)	50 Hz (continuous); 2 Hz (burst)	Paravertebral regions T10–L1 and S2–S4	Not specified	Not specified	Until the end of delivery
	100 Hz	Spinal nerve roots T10-L1 and S2-S4	Not specified	10–18 mA	30 min
Huang XZ 2019(66)	Self-adjusted	Bilateral Hegu (LI4), median nerve 4 cm proximal to wrist crease; proximal to wrist crease; highest point of iliac crest to lumbar spinous process	China Doule Group GT500 Series Seventh Generation Multi-functional DAOLE Doule Instrument	Mild muscle tremor induced	Self-adjusted usage time
R. Sulu 2022(61)	Not specified	Bilateral Zusanli (ST36), Hegu (LI4), Sanyinjiao (SP6)	Not specified	Gradually increased from 15 mA to obvious tremor	20 min/session, twice, 2-h interval, Stop when the cervix is fully dilated. M
Santana, L. S.2016(60)	Adjusted per tolerance	Hegu (LI4), Neiguan (PC6), paravertebral regions T10-L1 and S2-S4	SRL998A Bio-feed TENS System	Peak current: 15 mA	From the onset of the active phase until the end of the second stage of labor
Zahra MEHRI 2022(67)	2 Hz/100 Hz	Bilateral Hegu (LI4), Sanyinjiao (SP6), Zusanli (ST36)	Huatuo brand SDZ-II electronic acupuncture stimulator	Start at 15 mA, increase to slight muscle tremor and the pregnant woman’s tolerance level	Until the end of the second stage of labor
V. Rashtchi 2022(68)	2 Hz	Bilateral Hegu (LI4), Sanyinjiao (SP6), Zusanli (ST36)	Huatuo brand SDZ-II electronic acupuncture stimulator	Start at 15 mA, increase to painless	Every 2 h for 30 min, until active phase

#### 3.5.2 Duration of the first stage of labor

**Figure 5:**
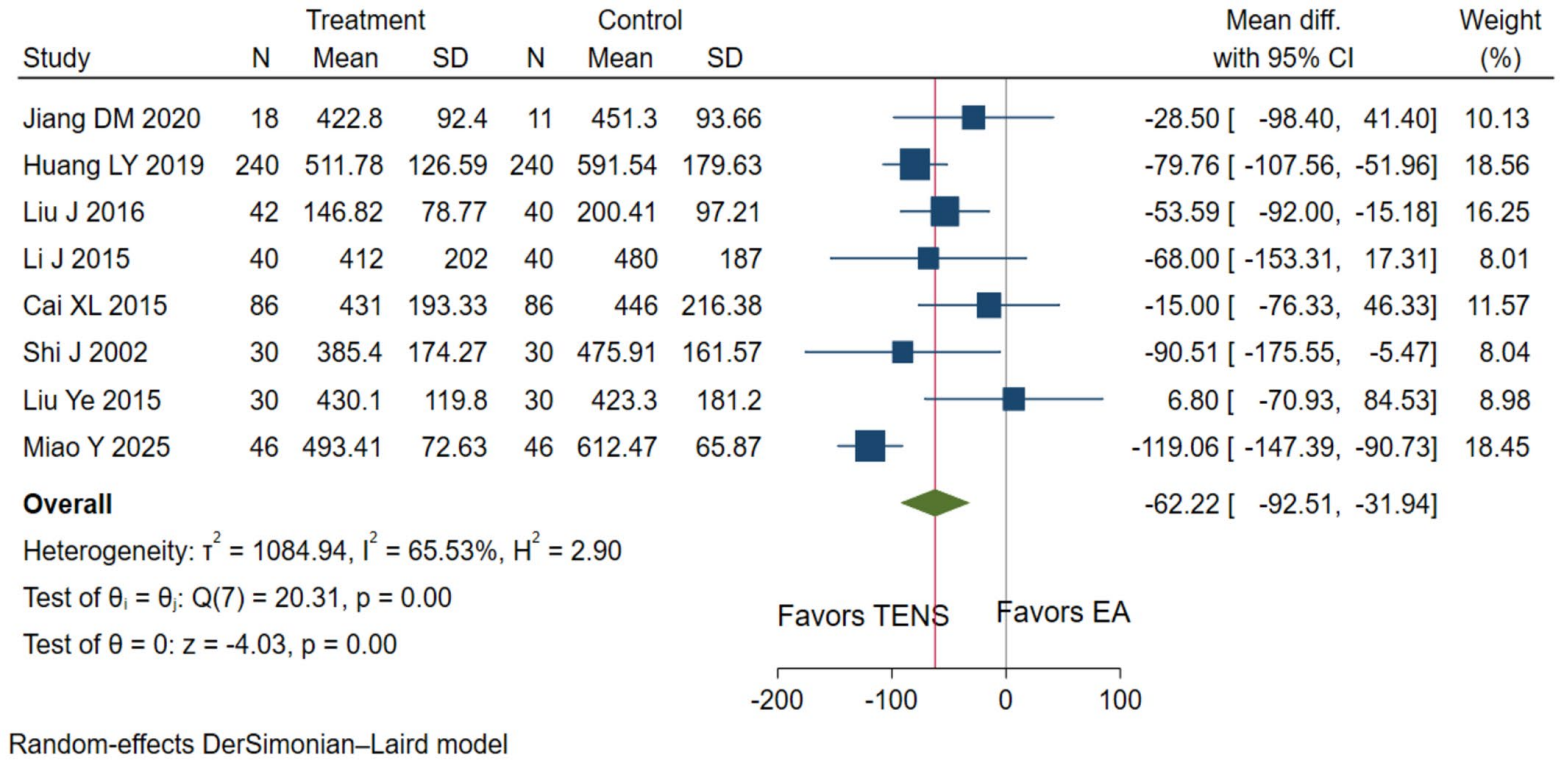
The duration of the first stage of labor with TENS analgesia was compared to epidural analgesia.

Low evidence (8 RCTs, 1,055 patients) reported that compared with epidural analgesia groups, parturients using TENS may shorten the duration of the first stage of labor (WMD-62.22 min, 95%CI –92.51 to −31.94 min; Table 3 and Figure 5) (28, 29, 34, 36, 37, 50, 54, 55, 57, 59).

#### 3.5.3 Adverse effects

**Figure 6:**
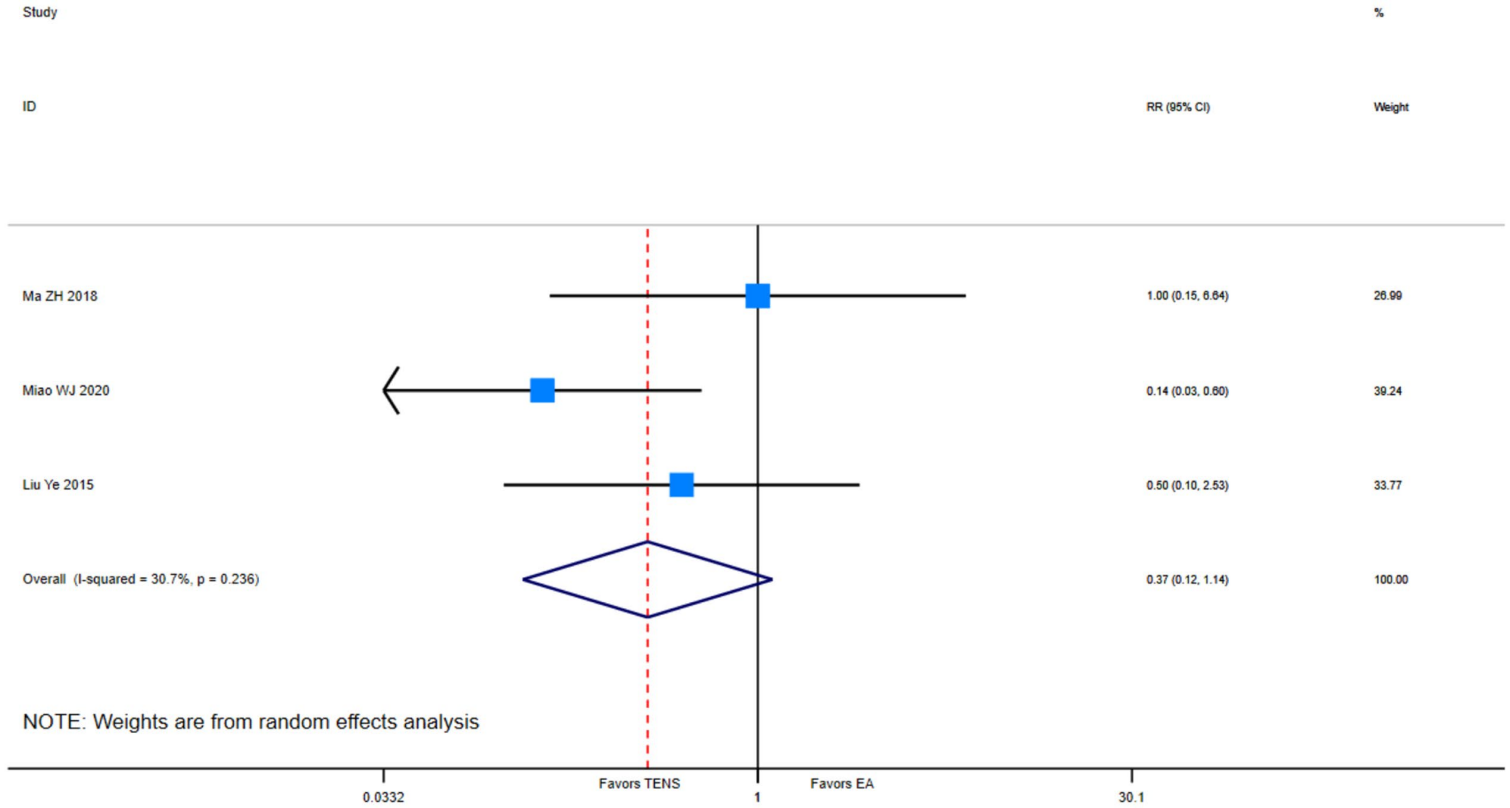
Adverse events among parturients during the first stage of labor who received TENS versus epidural analgesia.

Low evidence (3 RCTs, 271 patients) suggests that compared with epidural analgesia groups, parturients using TENS may be little to no difference in adverse effects (RR 0.37, 95%CI 0.12 to 1.14; Table 3 and Figure 6) (49, 50, 55).

## 4 Discussion

### 4.1 Overall findings

Low-quality evidence suggests that TENS may reduce pain intensity in the first stage of labor compared with sham treatment. When compared with epidural analgesia, TENS may shorten the duration of the first stage; however, no significant differences were observed in analgesic efficacy or the incidence of adverse effects.

### 4.2 Relations to other reviews

Despite four prior syntheses (75 trials total), one article was included. None of the studies exceeded 32 RCTs threshold surpassed by our 51-trial inclusion. The application of GRADE methodology in this review (absent in 75% of predecessors) provides critical evidence-level stratification.

### 4.3 Strengths and limitations

The primary strength of this review lies in its comprehensive consideration of two critical concerns during childbirth: effective pain management and labor duration reduction. However, several methodological limitations should be acknowledged, particularly concerning the elevated risk of bias in allocation concealment and blinding procedures.

### 4.4 Implications

This review holds significant clinical value by demonstrating that transcutaneous electrical nerve stimulation (TENS) serves as a viable alternative analgesic option for parturients who wish to avoid epidural analgesia during labor. Compared with no analgesic intervention, TENS has a pronounced pain-relieving effect. From a research perspective, our findings underscore the necessity for future clinical trials to optimize methodological rigor, particularly in allocation concealment and blinding procedures, to minimize potential bias.

## 5 Conclusion

In this systematic review and meta-analysis of randomized controlled trials on analgesia during the first stage of labor, low-certainty evidence suggests that, Compared with the blank control, TENS may reduce pain intensity and shorten the duration of the first stage of labor in parturients, with little to no difference in adverse events. Compared with epidural analgesia, TENS may shorten the duration of the first stage of labor, with no statistically significant differences observed in analgesic efficacy or adverse effects.

In summary, the current low-certainty evidence suggests that TENS may offer potential benefits for analgesia during the first stage of labor. However, to generate high-quality evidence applicable to clinical practice, there is an urgent need for future large-scale, methodologically rigorous randomized controlled trials that employ adequate randomization and blinding, utilize standardized intervention protocols, and report core clinical outcomes.

## Data Availability

The datasets presented in this study can be found in online repositories. The names of the repository/repositories and accession number(s) can be found in the article/Supplementary material.

## References

[1] Shi MCFX Hou JJ Ma X Zhou CX. Application progress of appropriate traditional Chinese medicine techniques in pain management during labor. J Nurs Sci. (2024) 39:20–4. doi: 10.3870/j.issn.1001-4152.2024.12.020

[2] Zhang MZG. Research Progress on the clinical application of labor analgesia. Jiangsu Medical J. (2020) 46:956–60. doi: 10.19460/j.cnki.0253-3685.2020.09.025

[3] Gu YYXF Li ZZ Zhang GY Gu CY. Transcutaneous electrical nerve stimulation for reducing labor pain in women undergoing trial of labor: a Meta-analysis. Chin. J. Women Child. Health. (2024) 15:63–72. doi: 10.19757/j.cnki.issn1674-7763.2024.06.010

[4] Nancy K Lowe P. The nature of labor pain. Am J Obstet Gynecol. (2002) 10.1067/mob.2002.121427 12011870

[5] Moher D Liberati A Tetzlaff J Altman DG. Preferred reporting items for systematic reviews and meta-analyses: the PRISMA statement. PLoS Med. (2009) 6:e1000097. doi: 10.1371/journal.pmed.1000097, 19621072 PMC2707599

[6] Yan W Kan Z Yin J Ma Y. Efficacy and safety of transcutaneous electrical Acupoint stimulation (TEAS) as An analgesic intervention for labor pain: a network Meta-analysis of randomized controlled trials. Pain Ther. (2023) 12:631–44. doi: 10.1007/s40122-023-00496-z, 36934401 PMC10199978

[7] Yan SX. Impact of epidural analgesia versus transcutaneous neuromuscular electrical stimulation on pregnancy and labor outcomes: A meta-analysis. Lanzhou University. (2019)

[8] Thuvarakan K Zimmermann H Mikkelsen MK Gazerani P. Transcutaneous electrical nerve stimulation as a pain-relieving approach in labor pain: a systematic review and Meta-analysis of randomized controlled trials. Neuromodulation. (2020) 23:732–46. doi: 10.1111/ner.13221, 32691942

[9] Melillo A Maiorano P Rachedi S Caggianese G Gragnano E Gallo L . Labor analgesia: a systematic review and Meta-analysis of non-pharmacological complementary and alternative approaches to pain during first stage of labor. Crit Rev Eukaryot Gene Expr. (2022) 32:61–89. doi: 10.1615/CritRevEukaryotGeneExpr.2021039986, 35381132

[10] Higgins JP Altman DG Gøtzsche PC Jüni P Moher D Oxman AD . The Cochrane collaboration's tool for assessing risk of bias in randomised trials. BMJ. (2011) 343:d5928. doi: 10.1136/bmj.d5928, 22008217 PMC3196245

[11] Akl EA Sun X Busse JW Johnston BC Briel M Mulla S . Specific instructions for estimating unclearly reported blinding status in randomized trials were reliable and valid. J Clin Epidemiol. (2012) 65:262–7. doi: 10.1016/j.jclinepi.2011.04.015, 22200346

[12] Thorlund K Walter SD Johnston BC Furukawa TA Guyatt GH. Pooling health-related quality of life outcomes in meta-analysis-a tutorial and review of methods for enhancing interpretability. Res Synth Methods. (2011) 2:188–203. doi: 10.1002/jrsm.46, 26061786

[13] Santesso N Glenton C Dahm P Garner P Akl EA Alper B . GRADE guidelines 26: informative statements to communicate the findings of systematic reviews of interventions. J Clin Epidemiol. (2020) 119:126–35. doi: 10.1016/j.jclinepi.2019.10.014, 31711912

[14] Wang Y Devji T Carrasco-Labra A King MT Terluin B Terwee CB . A step-by-step approach for selecting an optimal minimal important difference. BMJ. (2023) 381:e073822. doi: 10.1136/bmj-2022-073822, 37236647

[15] Page MJ HJ Sterne J. Chapter 13: Assessing risk of bias due to missing results in a synthesis. Cochrane Handbook for Systematic Reviews of Interventions version 64 (2023).

[16] Guyatt GH Oxman AD Vist GE Kunz R Falck-Ytter Y Alonso-Coello P . GRADE: an emerging consensus on rating quality of evidence and strength of recommendations. BMJ. (2008) 336:924–6. doi: 10.1136/bmj.39489.470347.AD, 18436948 PMC2335261

[17] Gao Y. Effect of transcutaneous low-frequency electrical stimulation combined with doula support on labor analgesia and outcomes in Primiparas. Lab Med Clin. (2023) 20:3514–7. doi: 10.3969/j.issn.1672-9455.2023.23.023

[18] Gao XSX Guo Y Lin L Han JX Huang M. Impact of low-frequency electrical nerve and muscle stimulation on labor Progress, delivery mode, and maternal-neonatal outcomes: An analysis. Progress Modern Biomed. (2021) 21:248–53. doi: 10.13241/j.cnki.pmb.2021.02.011

[19] Yan JSY. Effects of combined transcutaneous electrical nerve stimulation and epidural block for labor analgesia on maternal pain neurotransmitters, inflammatory cytokines, and stress factors. Maternal Child Health Care China. (2021) 36:955–8. doi: 10.19829/j.zgfybj.issn.1001-4411.2021.04.071

[20] Njogu A Qin S Chen Y Hu L Luo Y. The effects of transcutaneous electrical nerve stimulation during the first stage of labor: a randomized controlled trial. BMC Pregnancy Childbirth. (2021) 21:164. doi: 10.1186/s12884-021-03625-8, 33627077 PMC7905652

[21] Lei FYSJ Bi SJ Wang DM Han HJ Zheng DY Yu MJ. Efficacy and safety of transcutaneous electrical nerve stimulation combined with a birthing ball for labor analgesia: An investigation. *Sichuan*. J Tradit Chin Med. (2021) 39

[22] Peng LLLH Cai MY Wang Y Zhao Y Qi DM Luo CR. Effects of combined transcutaneous electrical nerve stimulation and epidural block on pain neurotransmitters, inflammatory factors, and stress response in Parturients undergoing labor analgesia. J Clin Experiment Med. (2021) 20:1760–3. doi: 10.3969/j.issn.1671-4695.2021.16.022

[23] Zhang XF. Efficacy of transcutaneous electrical nerve stimulation combined with intradermal sterile water injections for labor analgesia in Parturients: a study. Contemp Med Symp. (2020) 18:92–93.

[24] Zhang LQ. Application of transcutaneous electrical nerve stimulation in labor analgesia: a study. China Continu Med Educ. (2020) 12:120–2. doi: 10.3969/j.issn.1674-9308.2020.19.051

[25] Li HYCP Chen H Xu J. Evaluation of two analgesic interventions in doula-assisted delivery. Prev Med. (2020) 32:778–81. doi: 10.19485/j.cnki.issn2096-5087.2020.08.005

[26] Huang JZSL Li JS Wang ZH. Impact and safety analysis of combined electrical stimulation and doula analgesia on labor outcomes. J Binzhou Med Univers. (2020) 43:420–2. doi: 10.19739/j.cnki.issn1001-9510.2020.06.006

[27] Liu PPLJ Zhao YQ Zhang S Wang L. Effects of transcutaneous electrical nerve stimulation combined with birthing ball on labor Progress, labor pain, and analgesic drug utilization. Shanghai J Acupunct Moxibust. (2020) 39:47–52. doi: 10.13460/j.issn.1005-0957.2020.01.0047

[28] Jiang DMSH Lai XT Wang L Liu JH. Clinical application of doula support combined with transcutaneous electrical nerve stimulation in labor. Med Innov China. (2020) 17. doi: 10.3969/j.issn.1674-4985.2020.29.031

[29] Huang LYSP. Impact of full-course multimodal labor analgesia on maternal and neonatal safety. Maternal Child Health Care China. (2019) 34:5134–5137. doi: 10.7620/zgfybj.j.issn.1001-4411.2019.22.19

[30] Zhao ZPQL Lei ZW. Efficacy of Lamaze breathing combined with transcutaneous low-frequency electrical stimulation for labor analgesia and its impact on maternal-infant safety. Nurs Res. (2018) 32:3801–4. doi: 10.12102/j.issn.1009-6493.2018.23.041

[31] Lyu NLY Li L. Safety and efficacy of transcutaneous electrical nerve stimulation combined with epidural block for full-course labor analgesia. China Medical Herald. (2018) 15:94–97.

[32] Li LLY Zhai XJ Wang B Cui HY. Clinical study of transcutaneous electrical nerve stimulation for labor analgesia. Int J Obstetrics Gynecol. (2018) 45:37–40.

[33] Nyambura A. Application of transcutaneous electrical nerve stimulation therapy for pain relief during the first stage of labor. Hunan Normal University. (2020)

[34] Liu JQD Tan X Zuo L Qin JJ Li RM. Efficacy observation of transcutaneous electrical nerve stimulation for labor analgesia. J Jinan Univ. (2016) 37:416–9. doi: 10.11778/j.jdxb.2016.05012

[35] Xiao HWJ Wu MX. Adverse reactions assessment of combined electrical stimulation and epidural labor analgesia. (Natural Science & Medicine Edition). J Jinan Univ. (2015) 28:26. doi: 10.3969/j.issn.1006-1959.2015.11.032

[36] Li JYY. Study on biofeedback-based transcutaneous electrical stimulation for analgesia in natural childbirth. Shanxi Med J. (2015) 44:2113–5.

[37] Cai XLLD Yao JY Yuan YQ Feng M Jiang M. Impact of two analgesic modalities (transcutaneous electrical nerve stimulation and epidural anesthesia) under doula assistance on maternal delivery outcomes. Chin Foreign Med Res. (2015) 13:23–5. doi: 10.14033/j.cnki.cfmr.2015.24.013

[38] Xiao HWJ Kong JQ Li YQ. Clinical study of transcutaneous electrical nerve stimulation combined with epidural labor analgesia. J Clin Anesthesiol. (2014) 30:745–7.

[39] Li HYWZ Wang F Xu XF. Efficacy observation of transcutaneous electrical nerve stimulation for labor analgesia. Nurs Rehabil. (2012) 11:1140–1.

[40] Xu MJZG Chen L Zhang JY. Clinical study of HANS-assisted PCEA for Labor Analgesia. In: Peking University-Harvard Anesthesia and Pain Therapy Symposium (2006) 3 Beijing, China

[41] Su XJAJ. Clinical observation and evaluation of Han's Acupoint nerve stimulator (HANS) for labor analgesia. Chin J Pain Med. (2001) 2:89–93.

[42] Yang XCJ Hu LH. Effects of electrical stimulation at Hegu, Neiguan, and lumbosacral Acupoints on labor pain and duration in Parturients. Guangming J Chin Med. (2021) 36:2592–4. doi: 10.3969/j.issn.1003-8914.2021.15.043

[43] An ZZYZ Tong B Zhang YQ. Study on transcutaneous Acupoint electrical stimulation (TEAS) combined with Dexmedetomidine for labor analgesia. Shaanxi J Trad Chin Med. (2015) 36:1410–1.

[44] Cao JGZL Wang YF Li SH Luo W Wang CS. Effect of transcutaneous Acupoint electrical stimulation on intrapartum fever in Parturients receiving epidural labor analgesia. J Clin Anesthesiol. (2025) 41:36–39. doi: 10.12089/jca.2025.01.007

[45] Wang LLP. Effect of transcutaneous Acupoint electrical stimulation on labor pain perception in Primiparas undergoing natural childbirth. Electron J Pract Clin Nurs Sci. (2019) 4:30–1.

[46] Xu JH Xiong DD. Impact of transcutaneous Acupoint electrical stimulation-assisted remifentanil full-course intravenous labor analgesia on mother and infant. Pract Clin J Integrated Trad Chin Western Med. (2022) 22:6–9+13. doi: 10.13638/j.issn.1671-4040.2022.14.002

[47] Song KKYT Yang L Kong ZD Wang Q Gao W. Clinical efficacy of transcutaneous Acupoint electrical stimulation combined with epidural anesthesia for labor analgesia. Chongqing Med. (2023) 52:3133–6+41.

[48] He JWH Zhang RH. Application of transcutaneous Acupoint electrical stimulation combined with epidural block in painless labor and its impact on delivery outcomes. Shanghai J Acupunct Moxibust. (2020) 39:1269–73. doi: 10.13460/j.issn.1005-0957.2020.10.1269

[49] Ma ZHYF Zhao YY Jiao BJ Qu M Mao SH. Clinical study of transcutaneous Acupoint electrical stimulation combined with remifentanil for labor analgesia. Shanghai J Acupunct Moxibust. (2018) 37:526–30. doi: 10.13460/j.issn.1005-0957.2018.05.0526

[50] Miao WJ Qi WH Liu H. Role of Transcutaneous Acupoint Electrical Stimulation in Labor Analgesia. Chinese acupuncture and moxibustion. (2020) 40:615–618, 628. doi: 10.13703/j.0255-2930.20190824-0001 32538012

[51] Meng LKWM. Analgesic efficacy of Acupoint electrical stimulation combined with remifentanil PCIA for labor analgesia and its effect on peripheral blood β-endorphin levels. Psychol Monthly. (2020) 15:41+3. doi: 10.19738/j.cnki.psy.2020.01.028

[52] Han CPXW Xie QY. Comparison of analgesic efficacy between Acupoint electrical stimulation and Neuraxial anesthesia in multiparous women during labor. J Minimal Invasive Med. (2021) 16:51–3+62. doi: 10.11864/j.issn.1673.2021.01.11

[53] Zhao KLML Li Y Cui C Yang R Zhang L Li XY . Efficacy of combined epidural labor analgesia and transcutaneous Acupoint electrical stimulation in Primiparous women undergoing vaginal delivery. J Women and Children's Health Guide. (2024) 3

[54] Shi JWZ. Efficacy observation of analgesia device combined with tramadol and psychological nursing for labor analgesia. Nurs Res. (2002) 4:210–2. doi: 10.3969/j.issn.1009-6493.2002.04.014

[55] Liu Y Xu M Che X He J Guo D Zhao G . Effect of direct current pulse stimulating acupoints of JiaJi (T10-13) and Ciliao (BL 32) with Han's Acupoint nerve stimulator on labour pain in women: a randomized controlled clinical study. J Tradit Chin Med. (2015) 35:620–5. doi: 10.1016/s0254-6272(15)30149-7, 26742304

[56] Qian JCF Wang YX Yin MQ Zhu XQ. Application of transcutaneous electrical nerve stimulation in labor analgesia and its impact on maternal pain scores. Chin Sci J Database Med Health. (2025):57–60.

[57] Miao YLX Zhang X Li GL. Efficacy observation of epidural block anesthesia versus transcutaneous electrical nerve stimulation for painless delivery. J Bengbu Med Univ. (2025) 50:630–633. doi: 10.13898/j.cnki.issn.2097-5252.2025.05.015

[58] Xu JXM Lyu F Zhang N. Clinical observation of transcutaneous electrical nerve stimulation combined with epidural labor analgesia. China Med Herald. (2024) 21. doi: 10.2004/j.issn.1673-7210.2024.28.26

[59] Shi XLLL Shen T Gao Y. Efficacy of transcutaneous Acupoint electrical stimulation combined with continuous epidural anesthesia for labor analgesia in Primiparas. Pract Clin Med. (2024) 25:68–72. doi: 10.13764/j.cnki.lcsy.2024.04.018

[60] Santana LS Gallo RBS Ferreira CHJ Duarte G Quintana SM Marcolin AC. Transcutaneous electrical nerve stimulation (TENS) reduces pain and postpones the need for pharmacological analgesia during labour: a randomised trial. J Physiother. (2016) 62:29–34. doi: 10.1016/j.jphys.2015.11.002, 26701166

[61] Sulu R Akbas M Cetiner S. Effects of transcutaneous electrical nerve stimulation applied at different frequencies during labor on hormone levels, labor pain perception, and anxiety: a randomized placebo-controlled single-blind clinical trial. Eur J Integr Med. (2022) 52:102–124. doi: 10.1016/j.eujim.2022.102124

[62] Báez-Suárez A Martín-Castillo E García-Andújar J García-Hernández J Quintana-Montesdeoca MP Loro-Ferrer JF. Evaluation of different doses of transcutaneous nerve stimulation for pain relief during labour: a randomized controlled trial. Trials. (2018) 19:652. doi: 10.1186/s13063-018-3036-2, 30477529 PMC6258317

[63] Mehri Z Moafi F Alizadeh A Habibi M Ranjkesh F. Effect of acupuncture-like transcutaneous electrical nerve stimulation on labor pain in nulliparous women: a randomized controlled trial [J]. J Acu Tuina Sci. (2022) 20:376–382. doi: 10.1007/s11726-022-1298-4

[64] Movahedi M Ebrahimian M Saeedy M Tavoosi N. Comparative study of transcutaneous electrical nerve stimulation, the aromatherapy of Lavandula and physiologic delivery without medication on the neonatal and maternal outcome of patients. Int J Physiol Pathophysiol Pharmacol. (2022) 14:206–10. doi: 10.1007/s11726-022-1298-4 35891935 PMC9301182

[65] Niu CYTF Kong XL Yang HH. Application of low-frequency pulse electrical stimulation combined with traditional Chinese medicine Meridian theory in labor analgesia: a study. Chin J Mater Child Health Res. (2017) 28:43–4.

[66] H XZ. Clinical study of transcutaneous electrical nerve stimulation for labor analgesia. J Pract Med Techniq. (2019) 26:328–30. doi: 10.19522/j.cnki.1671-5098.2019.03.032

[67] Ranjkesh ZMA. Effect of acupuncture-like transcutaneous electrical nerve stimulation on labor pain in nulliparous women:a randomized controlled trial. J Acupunct Tuina Sci. (2022) 20:376–82. doi: 10.1007/s11726-022-1298-4

[68] Rashtchi V Maryami N Molaei B. Comparison of entonox and transcutaneous electrical nerve stimulation (TENS) in labor pain: a randomized clinical trial study. J Maternal-Fetal Neonatal Med. (2022) 35:3124–8. doi: 10.1080/14767058.2020.1813706, 32862743

