# Supplementary material for: The efficacy and safety of transcutaneous electrical nerve stimulation for labor analgesia in the first stage of labor: a qualitative and quantitative analysis
Source: Front Med (Lausanne). 2026 Jan 27;13:1730360. doi: 10.3389/fmed.2026.1730360 (PMC12888028; doi:10.3389/fmed.2026.1730360)
Supplement: Supplementary file 1 [file Table_1.docx]

**Supplement Table 1. Literature Search Strategies in Databases**

**Supplement Table 1.1 Pubmed search <inception to March 13, 2025>**

| **Search Strategy and Search Results** |
| --- |
| **Inception to October 17, 2024** |
| #1 Parturition[MeSH Terms] OR Pregnancy [MeSH Terms] OR Natural Childbirth[MeSH Terms] OR child labor[MeSH Terms] OR labor pain[MeSH Terms] OR labor analgesia[MeSH Terms] OR Analgesia, Obstetrical[MeSH Terms] OR labor onset[MeSH Terms] OR Delivery, Obstetric [MeSH Terms] OR Labor, Obstetric [MeSH Terms] OR Labor Stage, First[MeSH Terms]  (1151288)  #2 TI/AB:Parturition* OR Pregnancy* OR Pregnancies OR Childbirth* OR Birth* OR labor analgesia OR partus OR Labor OR Labor Onsets OR labor pain OR Obstetric Pain OR Obstetric Deliveries OR Obstetric Delivery OR Obstetric Labor OR Obstetrical Analgesia OR Obstetric Analgesia OR Analgesia* OR Gestation OR Water Birth* OR Lamaze Technique OR First Labor Stage OR First Stage Labor OR Cervical Dilatation* (1068092)  #3 #1 OR #2 163024  #4 transcutaneous electric nerve stimulation[MeSH Terms] OR transcutaneous nerve stimulation[MeSH Terms] OR electric stimulation[MeSH Terms] OR TENS [MeSH Terms] OR transdermal electrostimulation [MeSH Terms] OR percutaneous neuromodulation therapies[MeSH Terms] 141701  #5 TI/AB:transcutaneous electric nerve stimulation OR transcutaneous nerve stimulation OR electric stimulation OR TENS OR transdermal electrostimulation OR Transcutaneous Electric Stimulation OR percutaneous neuromodulation therapies OR Transcutaneous Electrical Stimulation OR Transdermal Electrostimulation OR Percutaneous Electrical Neuromodulation* OR Percutaneous Electrical Nerve Stimulation OR Transcutaneous Electrical Nerve Stimulation OR Transcutaneous Nerve Stimulation OR Percutaneous Neuromodulation Therapy OR Percutaneous Neuromodulation Therapies OR Electrical Neuromodulation, Percutaneous OR Electrical Neuromodulations, Percutaneous OR Analgesic Cutaneous Electrostimulation OR Electroanalgesia 30279  #6 #4 OR #5 165050  #7 Placebos[MeSH Terms]  #8 "Randomized Controlled Trial" or "Clinical Study" or randomized controlled trial OR controlled clinical trial OR placebo OR random* OR trial OR groups OR Clinical randomised controlled trial OR randomised control OR randomized OR RCT OR clinical trial  #9 #7 or #8 4307107  #10 #3 AND #6 AND #9 576 |
| **October 17, 2024 to July 22, 2025** |
| #1 Parturition[MeSH Terms] OR Pregnancy [MeSH Terms] OR Natural Childbirth[MeSH Terms] OR child labor[MeSH Terms] OR labor pain[MeSH Terms] OR labor analgesia[MeSH Terms] OR Analgesia, Obstetrical[MeSH Terms] OR labor onset[MeSH Terms] OR Delivery, Obstetric [MeSH Terms] OR Labor, Obstetric [MeSH Terms] OR Labor Stage, First[MeSH Terms]  (1181082)  #2 TI/AB:Parturition* OR Pregnancy* OR Pregnancies OR Childbirth* OR Birth* OR labor analgesia OR partus OR Labor OR Labor Onsets OR labor pain OR Obstetric Pain OR Obstetric Deliveries OR Obstetric Delivery OR Obstetric Labor OR Obstetrical Analgesia OR Obstetric Analgesia OR Analgesia* OR Gestation OR Water Birth* OR Lamaze Technique OR First Labor Stage OR First Stage Labor OR Cervical Dilatation* (1118966)  #3 #1 OR #2 1691960  #4 transcutaneous electric nerve stimulation[MeSH Terms] OR transcutaneous nerve stimulation[MeSH Terms] OR electric stimulation[MeSH Terms] OR TENS [MeSH Terms] OR transdermal electrostimulation [MeSH Terms] OR percutaneous neuromodulation therapies[MeSH Terms] （143227）  #5 TI/AB:transcutaneous electric nerve stimulation OR transcutaneous nerve stimulation OR electric stimulation OR TENS OR transdermal electrostimulation OR Transcutaneous Electric Stimulation OR percutaneous neuromodulation therapies OR Transcutaneous Electrical Stimulation OR Transdermal Electrostimulation OR Percutaneous Electrical Neuromodulation* OR Percutaneous Electrical Nerve Stimulation OR Transcutaneous Electrical Nerve Stimulation OR Transcutaneous Nerve Stimulation OR Percutaneous Neuromodulation Therapy OR Percutaneous Neuromodulation Therapies OR Electrical Neuromodulation, Percutaneous OR Electrical Neuromodulations, Percutaneous OR Analgesic Cutaneous Electrostimulation OR Electroanalgesia 32019  #6 #4 OR #5 168119  #7 Placebos[MeSH Terms]  #8 TI/AB:"Randomized Controlled Trial" or "Clinical Study" or randomized controlled trial OR controlled clinical trial OR placebo OR random* OR trial OR groups OR Clinical randomised controlled trial OR randomised control OR randomized OR RCT OR clinical trial  #9 #7 or #8 4551919  #10 AND (2024/10/28:2025/08/03[dp])  #11 #3 AND #6 AND #9#10 73 |

**Supplement Table 1.2 Embase search <inception to March 13, 2025>**

| **Search Strategy and Search Results** |
| --- |
| **Inception to October 17, 2024** |
| #1'transcutaneous electrical nerve stimulation'/exp  (10744)  #2 TI/AB/KW: 'electrostimulation, transcutaneous' OR 'nerve stimulation, transcutaneous' OR 'percutaneous electric nerve stimulation' OR 'percutaneous electrical nerve stimulation' OR 'tens (transcutaneous electrical nerve stimulation)' OR 'TENS ' OR 'transcutaneous electric nerve stimulation' OR 'transcutaneous electrical stimulation' OR 'transcutaneous electrostimulation' OR 'transcutaneous nerve stimulation' OR 'transcutaneous electrical nerve stimulation'  (25350)  #3 #1 OR #2 (31575)  #4 'obstetric analgesia'/exp OR 'birth'/exp OR 'labor induction'/exp OR 'pregnancy'/exp  (958112)  #5 TI/AB/KW: 'obstetric analgesia' OR 'analgesia, obstetric' OR 'analgesia, obstetrical' OR 'intravenous obstetric analgesia' OR 'intravenous obstetrical analgesia' OR 'labor analgesia' OR 'obstetric analgesia, intravenous' OR 'obstetrical analgesia' OR 'obstetrical analgesia, intravenous' OR 'birth' 'child birth' OR 'partus' OR 'childbirth' OR 'normal delivery' OR 'vaginal birth' OR 'vaginal childbirth' OR 'vaginal delivery' OR 'child bearing' OR 'childbearing' OR 'gestation' OR 'gravidity' OR 'intrauterine pregnancy' OR 'labor presentation' OR 'labour presentation' OR 'pregnancy maintenance' OR 'pregnancy trimesters' OR 'pregnancy' OR 'acceleration, labor' OR 'acceleration, labour' OR 'delivery induction' OR 'induced labor' OR 'induced labour' OR 'induction, delivery' OR 'induction, labor' OR 'induction, labour' OR 'labor acceleration' OR 'stimulated labor' OR 'labor induction' OR 'parturition' OR 'labor inducing' OR 'labor induction, premature' OR 'labor, induced' OR 'labor, stimulated' OR 'labour acceleration' OR 'labour inducing' OR 'labour induction' OR 'labour induction, premature' OR 'labour, induced' OR 'labour, stimulated' OR 'parturition induction' OR 'premature labor induction' (847715)  #6 #4 OR #5(1264354)  #7 'randomized controlled trial'/exp OR 'controlled trial, randomized' OR 'randomised controlled study' OR 'randomised controlled trial' OR 'randomized controlled study' OR 'trial, randomized controlled' OR 'randomized controlled trial' OR 'clinical trial'/exp OR 'clinical drug trial' OR 'major clinical trial' OR 'trial, clinical' OR 'clinical trial' OR 'placebo'/exp OR 'placebo gel' OR 'placebos' OR 'placebo' OR 'clinical study'/exp OR 'clinical data' OR 'clinical studies as topic' OR 'medical trial' OR 'clinical study'  (13760292)  #8 #3AND#6AND#7 283 |
| **October 17, 2024 to July 22, 2025** |
| #1('transcutaneous electrical nerve stimulation'/exp OR 'transcutaneous electrical nerve stimulation') AND [17-10-2024]/sd NOT [23-07-2025]/sd AND [2024-2025]/py  (690)  #2 ('electrostimulation, transcutaneous' OR 'nerve stimulation, transcutaneous' OR 'percutaneous electric nerve stimulation' OR 'percutaneous electrical nerve stimulation' OR 'tens (transcutaneous electrical nerve stimulation)' OR 'tens' OR 'transcutaneous electric nerve stimulation' OR 'transcutaneous electrical stimulation' OR 'transcutaneous electrostimulation' OR 'transcutaneous nerve stimulation' OR 'transcutaneous electrical nerve stimulation') AND [17-10-2024]/sd NOT [23-07-2025]/sd AND [2024-2025]/py  (1770)  #3 #1 OR #2 (1170)  #4 ('obstetric analgesia'/exp OR 'birth'/exp OR 'labor induction'/exp OR 'pregnancy'/exp) AND [17-10-2024]/sd NOT [23-07-2025]/sd AND [2024-2025]/py  (29274)  #5 TI/AB/KW:(('obstetric analgesia' OR 'analgesia, obstetric' OR 'analgesia, obstetrical' OR 'intravenous obstetric analgesia' OR 'intravenous obstetrical analgesia' OR 'labor analgesia' OR 'obstetric analgesia, intravenous' OR 'obstetrical analgesia' OR 'obstetrical analgesia, intravenous' OR 'birth') AND 'child birth' OR 'partus' OR 'childbirth' OR 'normal delivery' OR 'vaginal birth' OR 'vaginal childbirth' OR 'vaginal delivery' OR 'child bearing' OR 'childbearing' OR 'gestation' OR 'gravidity' OR 'intrauterine pregnancy' OR 'labor presentation' OR 'labour presentation' OR 'pregnancy maintenance' OR 'pregnancy trimesters' OR 'pregnancy' OR 'acceleration, labor' OR 'acceleration, labour' OR 'delivery induction' OR 'induced labor' OR 'induced labour' OR 'induction, delivery' OR 'induction, labor' OR 'induction, labour' OR 'labor acceleration' OR 'stimulated labor' OR 'labor induction' OR 'parturition' OR 'labor inducing' OR 'labor induction, premature' OR 'labor, induced' OR 'labor, stimulated' OR 'labour acceleration' OR 'labour inducing' OR 'labour induction' OR 'labour induction, premature' OR 'labour, induced' OR 'labour, stimulated' OR 'parturition induction' OR 'premature labor induction') AND [17-10-2024]/sd NOT [23-07-2025]/sd AND [2024-2025]/py (42113)  #6 #4 OR #5(43071)  #7('randomized controlled trial'/exp OR 'controlled trial, randomized' OR 'randomised controlled study' OR 'randomised controlled trial' OR 'randomized controlled study' OR 'trial, randomized controlled' OR 'randomized controlled trial' OR 'clinical trial'/exp OR 'clinical drug trial' OR 'major clinical trial' OR 'trial, clinical' OR 'clinical trial' OR 'placebo'/exp OR 'placebo gel' OR 'placebos' OR 'placebo' OR 'clinical study'/exp OR 'clinical data' OR 'clinical studies as topic' OR 'medical trial' OR 'clinical study') AND [17-10-2024]/sd NOT [23-07-2025]/sd AND [2024-2025]/py  (642094)  #8 #3AND#6AND#7 27 |
|  |

**Supplement Table 1.3 Cochrane search <inception to October 17, 2024>**

| **Search Strategy and Search Results** |
| --- |
| **inception to October 17, 2024** |
| #1TI/AB/KW ''Parturition*'' OR ''Childbirth*''OR ''Birth*'' OR ''Labor Pain' OR ''Obstetric Pain'' OR ''Analgesia, Obstetric*'' OR ''Obstetrical Analgesia'' OR ''labor onset*'' OR ''Obstetric Delivery'' OR ''labor Analgesia'' OR ''Obstetric Labor'' OR ''First Stage Labor'' OR ''Cervical Dilatation*''  (18498)  #2TI/AB/KW  ''Transcutaneous Electric Stimulation'' OR ''Transcutaneous Electrical Nerve Stimulation'' OR ''Transcutaneous Nerve Stimulation'' OR ''TENS'' OR ''Percutaneous Electrical Nerve Stimulation'' OR ''Transdermal Electrostimulation'' OR ''Percutaneous Electric Nerve Stimulation'' OR ''Transcutaneous Electrical Stimulation'' OR ''Analgesic Cutaneous Electrostimulation'' OR ''Electroanalgesia*'' OR ''Percutaneous Neuromodulation Therapies'' OR ''Neuromodulation*, Percutaneous Electrical'' OR ''Percutaneous Neuromodulation Therapy'' OR ''Percutaneous Electrical Neuromodulation*''  (42263)  #3 ''Randomized Controlled Trial'' OR ''Controlled Clinical Trials, Randomized'' OR ''Clinical Trials, Randomized'' OR ''Trials, Randomized Clinical'' OR ''Real World Clinical Trials'' OR ''Naturalistic Randomized Clinical Trial'' OR ''Practical Clinical Trials OR Trials, Practical Clinical OR Clinical Trials, Pragmatic'' OR ''Clinical Trials, Practical'' OR ''Trials, Pragmatic Clinical'' OR ''Pragmatic Clinical Trials'' OR ''Trials, Pragmatic'' OR ''Pragmatic Trials'' OR ''Clinical Trial'' OR ''Clinical study'' OR ''Placebo'' OR ''RCT'' (1825490)  #4 #1AND#2AND#3 438   \| **October 17, 2024 to July 22, 2025** \| \| --- \| \| #1TI/AB/KW ''Parturition*'' OR ''Childbirth*''OR ''Birth*'' OR ''Labor Pain' OR ''Obstetric Pain'' OR ''Analgesia, Obstetric*'' OR ''Obstetrical Analgesia'' OR ''labor onset*'' OR ''Obstetric Delivery'' OR ''labor Analgesia'' OR ''Obstetric Labor'' OR ''First Stage Labor'' OR ''Cervical Dilatation*''  (18918)  #2TI/AB/KW  ''Transcutaneous Electric Stimulation'' OR ''Transcutaneous Electrical Nerve Stimulation'' OR ''Transcutaneous Nerve Stimulation'' OR ''TENS'' OR ''Percutaneous Electrical Nerve Stimulation'' OR ''Transdermal Electrostimulation'' OR ''Percutaneous Electric Nerve Stimulation'' OR ''Transcutaneous Electrical Stimulation'' OR ''Analgesic Cutaneous Electrostimulation'' OR ''Electroanalgesia*'' OR ''Percutaneous Neuromodulation Therapies'' OR ''Neuromodulation*, Percutaneous Electrical'' OR ''Percutaneous Neuromodulation Therapy'' OR ''Percutaneous Electrical Neuromodulation*''  (43769)  #3 ''Randomized Controlled Trial'' OR ''Controlled Clinical Trials, Randomized'' OR ''Clinical Trials, Randomized'' OR ''Trials, Randomized Clinical'' OR ''Real World Clinical Trials'' OR ''Naturalistic Randomized Clinical Trial'' OR ''Practical Clinical Trials OR Trials, Practical Clinical OR Clinical Trials, Pragmatic'' OR ''Clinical Trials, Practical'' OR ''Trials, Pragmatic Clinical'' OR ''Pragmatic Clinical Trials'' OR ''Trials, Pragmatic'' OR ''Pragmatic Trials'' OR ''Clinical Trial'' OR ''Clinical study'' OR ''Placebo'' OR ''RCT'' (1446373)  #4 Between Oct,2024and Jul,2025  #5 #1AND#2AND#3AND#4 12 \| |

**Supplement Table 1.4 Web of Science search <inception to January 8, 2025>**

| **Search Strategy and Search Results** |
| --- |
| **Inception to October 17, 2024** |
| #1  (TS=(Parturition*) OR TS=( Pregnancy*) OR TS=(Natural Childbirth) OR TS=(child labor) OR TS=(labor pain) OR TS=( labor analgesia) OR TS=(Analgesia, Obstetrical) OR TS=(labor onset) OR TS=(Delivery, Obstetric ) OR TS=( Labor, Obstetric) OR TS=(Labor Stage, First) OR TS=(Pregnancies ) OR TS=(Childbirth*) OR TS=(Birth*) OR TS=(partus ) OR TS=(Labor) OR TS=(Labor Onset*) OR TS=(Obstetric Pain) OR TS=(Obstetric Deliveries) OR TS=(Obstetric Delivery) OR TS=(Obstetric Labor) OR TS=(Obstetric* Analgesia) OR TS=(Gestation) OR TS=(First Labor Stage) OR TS=(First Stage Labor) OR TS=(Cervical Dilatation*)) 1002368  #2 (TS=(transcutaneous electric nerve stimulation) OR TS=(transcutaneous nerve stimulation) OR TS=(electric stimulation) OR TS=(TENS) OR TS=(transdermal electrostimulation) OR TS=(percutaneous neuromodulation therapies) OR TS=(Transcutaneous Electric* Stimulation) OR TS=(Percutaneous Electrical Neuromodulation*) OR TS=(Percutaneous Electrical Nerve Stimulation) OR TS=(Electrical Neuromodulation, Percutaneous) OR TS=(Analgesic Cutaneous Electrostimulation) OR TS=(Electroanalgesia )) 84071  #3 (TS=(randomized controlled trial) OR TS=(randomized) OR TS=(placebo) OR TS=(randomised) OR TS=(random) OR TS=(RCT) OR TS=(clinical trial) OR TS=(clinical study) OR TS=(trial)) 7044458  #5 #1 AND #2 AND #3 AND #4 333 |
| **October 17, 2024 to July 22, 2025** |
| #1 ((TS=(Polycystic Ovary Syndrome) OR TS=(Ovary Syndrome, Polycystic) OR TS=(Syndrome, Polycystic Ovary) OR TS=(Stein-Leventhal Syndrome) OR TS=(Stein Leventhal Syndrome) OR TS=(Syndrome, Stein-Leventhal) OR TS=(Sclerocystic Ovarian Degeneration) OR TS=(Ovarian Degeneration, Sclerocystic) OR TS=(Sclerocystic Ovary Syndrome) OR TS=(Polycystic Ovarian Syndrome) OR TS=(Ovarian Syndrome, Polycystic) OR TS=(Polycystic Ovary Syndrome 1) OR TS=(Sclerocystic Ovaries) OR TS=(Ovary, Sclerocystic) OR TS=(Sclerocystic Ovary) OR TS=(cystic ovary) OR TS=(micropolycystic ovary) OR TS=(multiple follicle cyst) OR TS=(PCOS) OR TS=(PCOD) OR TS=(ovary polycystic disease) OR TS=(Hyperandrogenism) OR TS=(hyper-androgenism) OR TS=(hyperandro-genism) OR TS=(Hyperandrogenaemia) OR TS=(Hyperandrogenemia) OR TS=(Hyperandrogenism) OR TS=(Hirsutism) OR TS=(Acne) OR TS=(Amenorrhea) OR TS=(amenorrhoea) OR TS=(menstrual retention) OR TS=(amenorrhea) OR TS=(Amenorrhea) OR TS=(Oligomenorrhea) OR TS=(ovulatory) OR TS=(Dysfunction) OR TS=(Anovulation) OR TS=(Infertility) OR TS=(Androgens) OR TS=(Virilism))) Last 5 years (26625)  #2 (TS=(Obesity) OR TS=(Overweight) OR TS=(Body Mass Index) OR TS=(adiposity) OR TS=(Quetelet Index) OR TS=(Index, Quetelet) OR TS=(Quetelet's Index) OR TS=(Quetelets Index) OR TS=(Index, Body Mass) OR TS=(polysarcia) OR TS=(corpulent) OR TS=(BMI) OR TS=(obese) OR TS=(weight loss) OR TS=(weight control) OR TS=(weight reduction) OR TS=(Body Fat Distribution)) Last 5 years(415728)  #3 (TS=(catgut embedding) OR TS=(acupoint catgut embedding) OR TS=(catgut implantation) OR TS=(embedding thread) OR TS=(point embedding therapy) OR TS=(Acupoint Ligat Thread Embed) OR TS=(Embedding Therapy) OR TS=(acupuncture therapy) OR TS=(Acupoint Therapy) OR TS=(Acupuncture Point*) OR TS=(Acupuncture) OR TS=(Acupoint*)) Last 5 years (13410)  #4 (TS=(randomized controlled trial) OR TS=(randomized) OR TS=(placebo) OR TS=(randomised) OR TS=(random) OR TS=(RCT) OR TS=(clinical trial) OR TS=(clinical study) OR TS=(trial)) Last 5 years (1508412)  #5 #1 AND #2 AND #3 AND #4 (99) |

**Supplement Table 1.5 CNKI search <inception to October 17, 2024>**

| **Search Strategy and Search Results** |
| --- |
| **inception to October 17, 2024** |
| #1 Title/Abstract/Keywords: epidural analgesia OR labor pain OR painless labor OR obstetric analgesia OR childbirth OR vaginal delivery OR parturition OR trial of labor after cesarean  #2 Title/Abstract/Keywords: transcutaneous electric nerve stimulation OR electrical nerve stimulation OR percutaneous electrical stimulation OR electrostimulation  #3 Title/Abstract/Keywords: random* OR control* OR randomized controlled trial OR placebo OR clinical trial  #4 #1 AND #2 AND #3  (704) |
| **October 17, 2024 to July 22, 2025** |
| #1 Title/Abstract/Keywords: epidural analgesia OR labor pain OR painless labor OR obstetric analgesia OR childbirth OR vaginal delivery OR parturition OR trial of labor after cesarean  #2 Title/Abstract/Keywords: transcutaneous electric nerve stimulation OR electrical nerve stimulation OR percutaneous electrical stimulation OR electrostimulation  #3 Title/Abstract/Keywords: random* OR control* OR randomized controlled trial OR placebo OR clinical trial  #4 Time range limited from October 17, 2024 to July 22, 2025  #5 #1AND#2AND#3AND#4  （31） |

**Supplement Table 1.6 Wan Fang search <inception to October 17, 2024>**

| **Search Strategy and Search Results** |
| --- |
| **inception to October 17, 2024** |
| #1 Title/Abstract/Keywords: epidural analgesia OR labor pain OR painless labor OR obstetric analgesia OR childbirth OR vaginal delivery OR parturition OR trial of labor  #2 Title/Abstract/Keywords: transcutaneous electric nerve stimulation OR electrical nerve stimulation OR percutaneous electrical stimulation OR electrostimulation  #3 Abstract: random* OR control* OR randomized controlled trial OR placebo OR clinical trial  #4 #1AND#2AND#3  (610) |
| **October 17, 2024 to July 22, 2025** |
| #1 Title/Abstract/Keywords: epidural analgesia OR labor pain OR painless labor OR obstetric analgesia OR childbirth OR vaginal delivery OR parturition OR trial of labor  #2 Title/Abstract/Keywords: transcutaneous electric nerve stimulation OR electrical nerve stimulation OR percutaneous electrical stimulation OR electrostimulation  #3 Abstract: random* OR control* OR randomized controlled trial OR placebo OR clinical trial  #4 2024-2025 time period  #5 #1AND#2AND#3AND#4  (45) |

**Supplement Table 1.7VIP search <inception to October 17, 2024>**

| **Search Strategy and Search Results** |
| --- |
| **inception to October 17, 2024** |
| Search Query:  ((M=(epidural analgesia OR labor pain OR painless labor OR obstetric analgesia OR vaginal delivery OR childbirth OR parturition OR trial of labor)) OR (R=(epidural analgesia OR labor pain OR painless labor OR obstetric analgesia OR vaginal delivery OR childbirth OR parturition OR trial of labor))) AND ((M=(percutaneous electrical stimulation OR electrical nerve stimulation OR transcutaneous electrical nerve stimulation OR electrical stimulation)) OR (R=(percutaneous electrical stimulation OR electrical nerve stimulation OR transcutaneous electrical nerve stimulation OR electrical stimulation))) AND ((M=(random* OR control* OR randomized controlled trial OR placebo OR clinical trial)) OR (R=(random* OR control* OR randomized controlled trial OR placebo OR clinical trial)))  (604) |
| **October 17, 2024 to July 22, 2025** |
| Search Query:  ((M=(epidural analgesia OR labor pain OR painless labor OR obstetric analgesia OR vaginal delivery OR childbirth OR parturition OR trial of labor)) OR (R=(epidural analgesia OR labor pain OR painless labor OR obstetric analgesia OR vaginal delivery OR childbirth OR parturition OR trial of labor))) AND ((M=(percutaneous electrical stimulation OR electrical nerve stimulation OR transcutaneous electrical nerve stimulation OR electrical stimulation)) OR (R=(percutaneous electrical stimulation OR electrical nerve stimulation OR transcutaneous electrical nerve stimulation OR electrical stimulation))) AND ((M=(random* OR control* OR randomized controlled trial OR placebo OR clinical trial)) OR (R=(random* OR control* OR randomized controlled trial OR placebo OR clinical trial)))2024-2025 time period  (70) |
