# Supplementary material for: The efficacy and safety of transcutaneous electrical nerve stimulation for labor analgesia in the first stage of labor: a qualitative and quantitative analysis
Source: Front Med (Lausanne). 2026 Jan 27;13:1730360. doi: 10.3389/fmed.2026.1730360 (PMC12888028; doi:10.3389/fmed.2026.1730360)
Supplement: Supplementary file 2 [file Table_2.docx]

**Supplement Table 2. Risk bias of included studies**

| **Study** | **Adequate randomization sequence generation** | **Adequate allocation concealment** | **Blinding of patients** | **Blinding of health care providers** | **Blinding of data collectors** | **Blinding of outcome assessors** | **Blinding of data analyst** | **Loss to follow-up (%)** |
| --- | --- | --- | --- | --- | --- | --- | --- | --- |
| Gao Y 2023 | High | High | High | High | High | High | High | 0 |
| M. Movahedi 2022 | Low | High | High | High | High | High | High | 0 |
| Gao XX 2021 | High | High | High | High | High | High | High | 5% |
| Yan J 2021 | Low | High | High | High | High | High | High | 0 |
| A. Njogu 2021 | Low | Low | Low | High | High | High | Low | 5.8% |
| Lei FY 2021 | Low | High | High | High | High | High | High | 7.14% |
| Peng LL 2021 | Low | High | High | High | High | High | High | 0 |
| Zhang XF 2020 | High | High | High | High | High | High | High | 0 |
| Zhang LQ 2020 | High | High | High | High | High | High | High |  |
| Li HY 2020 | Low | High | High | High | High | High | High | 12.36% |
| Huang JZ 2020 | High | High | High | High | High | High | High | 0 |
| Liu PP 2020 | Low | High | High | High | High | High | High | 0 |
| Jiang DM 2020 | Low | High | High | High | High | High | High | 42% |
| Huang LY 2019 | Low | High | High | High | High | High | High | 0 |
| Zhao ZP 2018 | Low | High | High | High | High | High | High | 0 |
| A. Baez-Suarez 2018 | Low | Low | Low | Low | High | Low | High | 0 |
| Lu L 2018 | Low | High | High | High | High | High | High | 11% |
| Li L 2018 | High | High | High | High | High | High | High | 0 |
| Liu CY 2017 | High | High | High | High | High | High | High | 16% |
| A.Nyambura 2017 | High | High | High | High | High | High | High | 0 |
| Liu J 2016 | Low | High | High | High | High | High | High | 19.5% |
| Li J 2015 | Low | High | High | High | High | High | High | 0 |
| Cai XL 2015 | High | High | High | High | High | High | High | 0 |
| Xiao H 2015 | High | High | High | High | High | High | High | 25% |
| Li HY 2012 | Low | High | High | High | High | High | High | 0 |
| Xu MJ 2006 | High | High | High | High | High | High | High | 15.83% |
| Su XJ 2001 | High | High | High | High | High | High | High | 0 |
| Yang X 2021 | High | High | High | High | High | High | High | 0 |
| An ZZ 2015 | High | High | High | High | High | High | High | 0 |
| Cao JG 2025 | Low | High | High | High | High | High | High | 0 |
| Wang L 2019 | Low | High | High | High | High | High | High | 0 |
| Xu JH 2022 | Low | High | High | High | High | High | High | 0 |
| Song KK 2023 | Low | High | High | High | High | High | High | 0 |
| He J 2020 | Low | High | High | High | High | High | High | 0 |
| Ma ZH 2018 | Low | High | High | High | High | High | High | 0 |
| Miao WJ 2020 | Low | High | High | High | High | High | High | 0 |
| Meng LK 2020 | Low | High | High | High | High | High | High | 0 |
| Han CP 2021 | High | High | High | High | High | High | High | 0 |
| Zhao KL 2024 | Low | High | High | High | High | High | High | 0 |
| Shi J 2002 | High | High | High | High | High | High | High | 0 |
| Liu Ye 2015 | Low | High | High | High | High | High | High | 0 |
| QianJ 2025 | Low | High | High | High | High | High | High | 0 |
| Miao Y 2025 | High | High | High | High | High | High | High | 0 |
| Xu J 2024 | Low | High | High | High | High | High | High | 0 |
| Shi XL 2024 | Low | High | High | High | High | High | High | 0 |
| Huang XZ 2019 | Low | High | High | High | High | High | High | 0 |
| R. Sulu 2022 | Low | Low | Low | High | High | High | High | 0 |
| Santana, L. S.2016 | Low | Low | High | High | High | Low | High | 0 |
| Zahra MEHRI 2022 | Low | High | High | High | High | High | High | 9.7% |
| V. Rashtchi 2022 | Low | High | High | High | High | High | High | 0 |
