# Supplementary material for: The efficacy and safety of transcutaneous electrical nerve stimulation for labor analgesia in the first stage of labor: a qualitative and quantitative analysis
Source: Front Med (Lausanne). 2026 Jan 27;13:1730360. doi: 10.3389/fmed.2026.1730360 (PMC12888028; doi:10.3389/fmed.2026.1730360)
Supplement: Supplementary file 3 [file Table_3.docx]

| **Reference ID** | **RCT** | **Reasons to exclude** |
| --- | --- | --- |
| 1 | A. Aghamohammadi 2013 | ineligible outcome |
| 2 | Bastani FF 2020 | full text not available |
| 3 | Chao AS 2007 | full text not available |
| 4 | Chestnut DH 1990 | ineligible outcome |
| 5 | Kamali A 2018 | ineligible intervention |
| 6 | Liu Y 2015 | Included |
| 7 | Mehri Z 2022 | full text not available |
| 8 | Miao WJ 2020 | Included |
| 9 | Mucuk S | full text not available |
| 10 | Shen X | ineligible intervention |
| 11 | Li XR 2013 | ineligible intervention |
| 12 | Wang HH | ineligible intervention |
| 13 | Ren XY 2015 | ineligible intervention |
| 14 | Li XR 2015 | ineligible intervention |
| 15 | Wei DH 2013 | ineligible intervention |
| 16 | Yang YX 2015 | ineligible intervention |
| 17 | Deng Q 2016 | ineligible intervention |
| 18 | Li J 2007 | ineligible outcome |
| 19 | . Santana LS 2016 | ineligible outcome |
| 20 | . Dowswell T 2009 | full text not available |
| 21 | Aghamohammadi A 2011 | full text not available |
| 22 | Báez-Suárez A 2018 | Included |
| 23 | Borup L 2009 | ineligible outcome |
| 24 | . Bundsen P, 1982 | full text not available |
| 25 | Chao AA-S 2007 | full text not available |
| 26 | Chia YT 1990 | full text not available |
| 27 | . Dong C, | ineligible intervention |
| 28 | Erkkola R 1980 | ineligible outcome |
| 29 | Harrison RF 1986 | ineligible outcome |
| 30 | Harrison RF 1987 | ineligible outcome |
| 31 | .Labrecque M 1999 | ineligible intervention |
| 32 | Lee EW 1990 | ineligible outcome |
| 33 | Liu Ye 2015 | Included |
| 34 | Nesheim BI 1981 | full text not available |
| 35 | . Padma, A 2000 | ineligible outcome |
| 36 | Thakur R 2004 | ineligible intervention |
| 37 | Samadzadeh S 2017 | ineligible intervention |
| 38 | Shahoei R 2017 | full text not available |
| 39 | Steptoe P 1984 | full text not available |
| 40 | Tawfik O 1982 | ineligible outcome |
| 41 | Thomas IL 1988 | full text not available |
| 42 | Tsen LC 2000 | ineligible outcome |
| 43 | Van der Ploeg JM 1996 | ineligible outcome |
| 44 | Wang B 2006 | ineligible outcome |
| 45 | Wu Q 2021 | ineligible intervention |
| 46 | Niu CY 2021 | Included |
| 47 | Mehri Z 2022（重复） | full text not available |
| 48 | Gao Y 2023 | Included |
| 49 | Chen Y 2022 | non-randomized |
| 50 | Lei FY 2021 | Included |
| 51 | Zhou J 2021 | ineligible intervention |
| 52 | Zhu Y 2021 | non-randomized |
| 53 | Grymel KE 2021 | ineligible intervention |
| 54 | Movahedi M 2022 | Included |
| 55 | Njogu A 2021 | Included |
| 56 | Baez SA 2018 | Included |
| 57 | Chao AS 2007（重复） | full text not available |
| 58 | Czech I 2018 | ineligible outcome |
| 59 | Dong C 2015 | ineligible intervention |
| 60 | Rashtchi V 2022 | ineligible intervention |
| 61 | Sever N 2015 | full text not available |
| 62 | Shahoei R 2017（重复） | full text not available |
| 63 | Liu PP 2020 | Included |
| 64 | Cui YF 2018 | full text not available |
| 65 | Zhang LQ 2020 | Included |
| 66 | Zhang CH 2015 | ineligible intervention |
| 67 | Li J 2015 | Included |
| 68 | Li HY 2012 | Included |
| 69 | Li HY 2020 | Included |
| 70 | Li L 2018 | Included |
| 71 | Huang XZ 2019 | ineligible outcome |
| 72 | Huang T 2008 | non-randomized |
| 73 | Wang SS 2019 | non-randomized |
| 74 | Qiu SF 2015 | Included |
| 75 | Huang JZ 2020 | Included |

**Supplement Table 3. The reasons to exclude included studies of previous systematic reviews of ACE for PCOS**
