# Supplementary figures and images for: The efficacy and safety of transcutaneous electrical nerve stimulation for labor analgesia in the first stage of labor: a qualitative and quantitative analysis
Source: Front Med (Lausanne). 2026 Jan 27;13:1730360. doi: 10.3389/fmed.2026.1730360 (PMC12888028; doi:10.3389/fmed.2026.1730360)

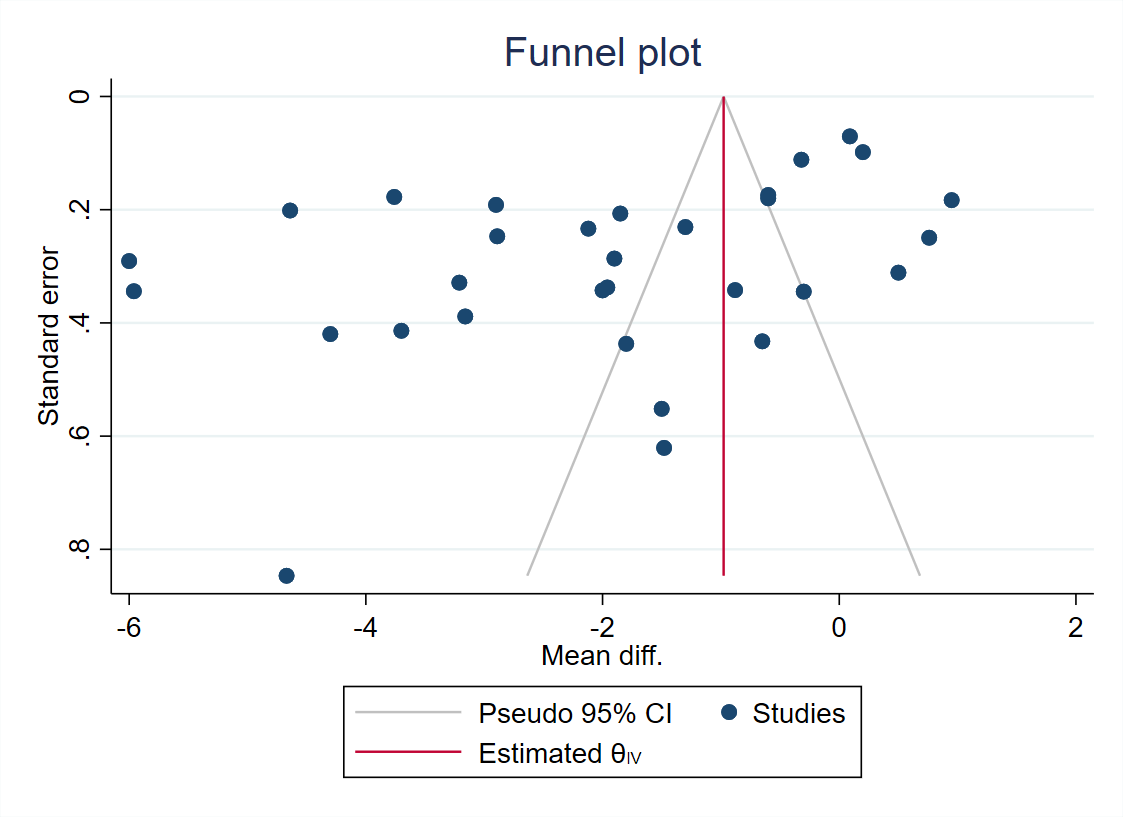


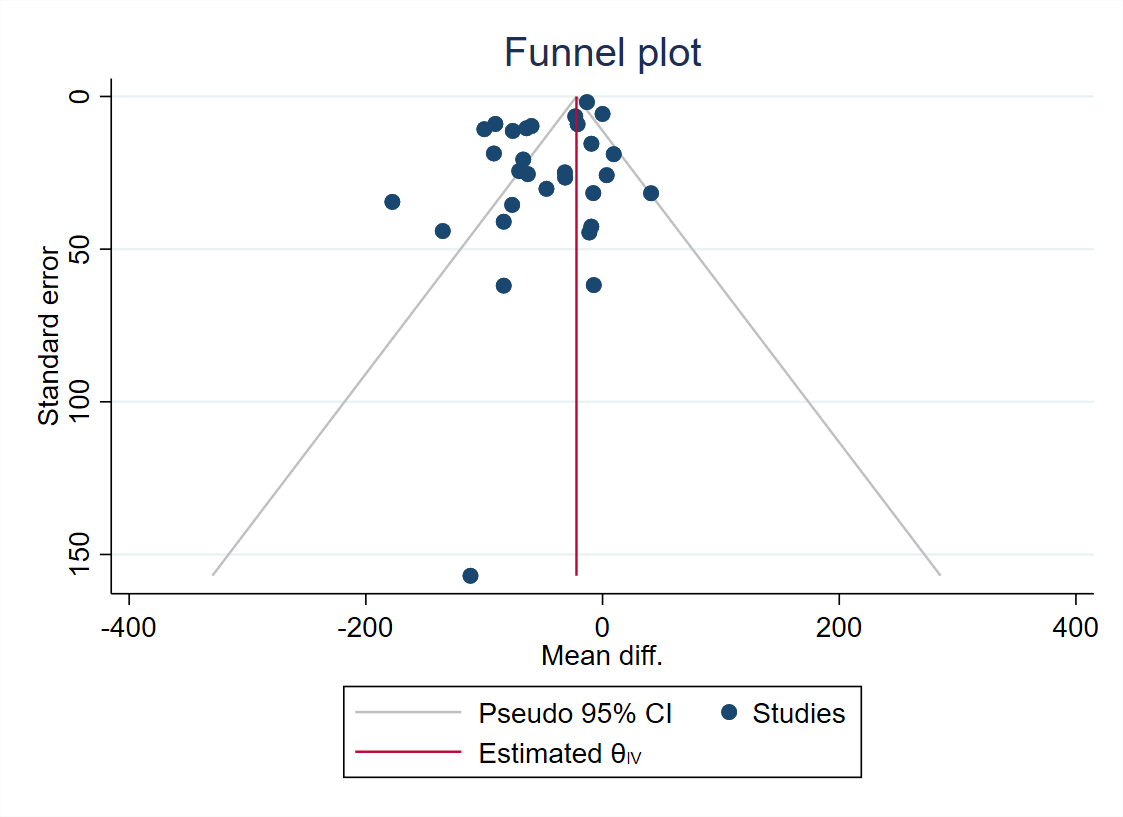


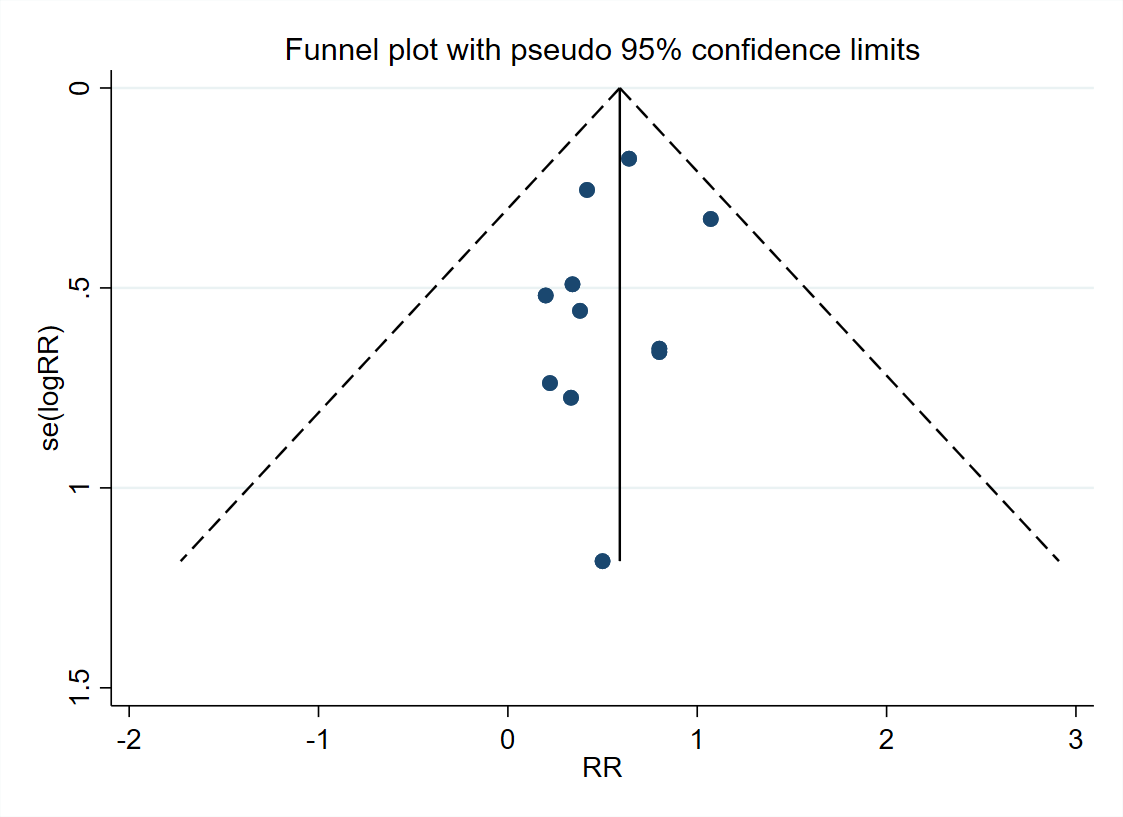

Supplement: Supplementary file 4 [file Data_Sheet_1.docx]
